# Cooperative Blockade of CK2 and ATM Kinases Drives Apoptosis in VHL-Deficient Renal Carcinoma Cells through ROS Overproduction

**DOI:** 10.3390/cancers13030576

**Published:** 2021-02-02

**Authors:** Sofia Giacosa, Catherine Pillet, Irinka Séraudie, Laurent Guyon, Yann Wallez, Caroline Roelants, Christophe Battail, Bertrand Evrard, Frédéric Chalmel, Caroline Barette, Emmanuelle Soleilhac, Marie-Odile Fauvarque, Quentin Franquet, Clément Sarrazin, Nicolas Peilleron, Gaëlle Fiard, Jean-Alexandre Long, Jean-Luc Descotes, Claude Cochet, Odile Filhol

**Affiliations:** 1 Interdisciplinary Research Institute of Grenoble, IRIG-Biosanté, University Grenoble Alpes, CEA, UMR 1292, F-38000 Grenoble, France; sofia.giacosa@ext.erytech.com (S.G.); catherine.pillet@cea.fr (C.P.); irinka.seraudie2@cea.fr (I.S.); laurent.guyon@cea.fr (L.G.); yann.wallez@astrazeneca.com (Y.W.); christophe.battail@cea.fr (C.B.); caroline.barette@cea.fr (C.B.); emmanuelle.soleilhac@cea.fr (E.S.); marie-odile.fauvarque@cea.fr (M.-O.F.); qfranquet@chu-grenoble.fr (Q.F.); csarrazin1@chu-grenoble.fr (C.S.); nicolas.peilleron@gmail.com (N.P.); claude.cochet@cea.fr (C.C.); 2 Bioscience, Early Oncology R&D, AstraZeneca, Cambridge CB2 1TN, UK; 3 Inovarion, F-75005 Paris, France; caroline.roelants@inovarion.com; 4 Inserm, EHESP, Irset (Institut de Recherche en Santé, Environnement et Travail), University Rennes, UMR_S 1085, F-35000 Rennes, France; bertrand.evrard@univ-rennes1.fr (B.E.); frederic.chalmel@univ-rennes1.fr (F.C.); 5 Centre Hospitalier Universitaire Grenoble Alpes, CS 10217, CEDEX 9, F-38043 Grenoble, France; gfiard@chu-grenoble.fr (G.F.); JALong@chu-grenoble.fr (J.-A.L.); jldescotes@chu-grenoble.fr (J.-L.D.); 6 TIMC-IMAG, Grenoble Institut National Polytechnique, CNRS, University Grenoble Alpes, F-38000 Grenoble, France

**Keywords:** ccRCC, ATM, CK2, kinase inhibitor, HIF-2α, ROS pathway, NOX4, apoptosis, tumor tissue slices

## Abstract

**Simple Summary:**

Renal cell carcinoma (RCC) is the eighth leading malignancy in the world, accounting for 4% of all cancers with poor outcome when metastatic. Protein kinases are highly druggable proteins, which are often aberrantly activated in cancers. The aim of our study was to identify candidate targets for metastatic clear cell renal cell carcinoma therapy, using chemo-genomic-based high-throughput screening. We found that the combined inhibition of the CK2 and ATM kinases in renal tumor cells and patient-derived tumor samples induces synthetic lethality. Mechanistic investigations unveil that this drug combination triggers apoptosis through HIF-2α-(Hypoxic inducible factor HIF-2α) dependent reactive oxygen species (ROS) overproduction, giving a new option for patient care in metastatic RCC.

**Abstract:**

Kinase-targeted agents demonstrate antitumor activity in advanced metastatic clear cell renal cell carcinoma (ccRCC), which remains largely incurable. Integration of genomic approaches through small-molecules and genetically based high-throughput screening holds the promise of improved discovery of candidate targets for cancer therapy. The 786-O cell line represents a model for most ccRCC that have a loss of functional pVHL (von Hippel-Lindau). A multiplexed assay was used to study the cellular fitness of a panel of engineered ccRCC isogenic 786-O VHL^−^ cell lines in response to a collection of targeted cancer therapeutics including kinase inhibitors, allowing the interrogation of over 2880 drug–gene pairs. Among diverse patterns of drug sensitivities, investigation of the mechanistic effect of one selected drug combination on tumor spheroids and ex vivo renal tumor slice cultures showed that VHL-defective ccRCC cells were more vulnerable to the combined inhibition of the CK2 and ATM kinases than wild-type VHL cells. Importantly, we found that HIF-2α acts as a key mediator that potentiates the response to combined CK2/ATM inhibition by triggering ROS-dependent apoptosis. Importantly, our findings reveal a selective killing of VHL-deficient renal carcinoma cells and provide a rationale for a mechanism-based use of combined CK2/ATM inhibitors for improved patient care in metastatic VHL-ccRCC.

## 1. Introduction

Renal cell carcinoma (RCC) is the eighth leading malignancy in the world, accounting for 4% of all cancers. RCC patients do not show obvious pathognomonic symptoms but approximately 30% of them present with metastatic disease at the time of diagnosis and nearly half of the remainder will subsequently develop metastasis (mRCC) [1]. Clear cell renal cell carcinoma (ccRCCs) is the most common form of kidney cancer, accounting for 70–90% of all cases [2]. The poor outcome of ccRCCs is due to the heterogeneous and aggressive nature of the disease, coupled with the lack of actionable biomarkers that can be used to direct therapy [3,4]. Therapeutic response in patients with clinically comparable tumors may differ significantly. Consequently, one of the key challenges in ccRCC is to define tangible means for patient stratification and to delineate targeted approaches to treatment. Most ccRCC have somatic mutations of both alleles of pVHL (von Hippel-Lindau), a component of an E3 ubiquitin ligase complex, which targets the hypoxia-inducible factors (HIF), HIF-1α and HIF-2α, for degradation [5]. Inactivation of pVHL in ccRCC leads to the accumulation of HIF-2α [6], and reintroduction of a pVHL protein in VHL-deficient ccRCC cells downregulates HIF-2α and suppresses their ability to form tumors [7]. The frequency of HIF accumulation is a critical oncogenic feature representing a potentially relevant event for treatment stratification [5,7,8,9,10,11]. Under normoxic conditions, the oxygen-sensitive α subunit of HIF-1 and HIF-2 are hydroxylated on two proline residues by the oxygen-dependent HIFα-specific prolyl hydroxylases and degraded by VHL-mediated ubiquitination. However, under hypoxic conditions, HIFα degradation is suppressed, leading to enhanced nuclear localization of HIFα and transcription of various target genes, including the angiogenic factor gene for vascular endothelial growth factor (VEGF) and other genes involved in tumor progression and metastasis [12]. 

Protein kinases—a highly druggable class of proteins—are often aberrantly activated in cancers and participate in the development of resistance to current treatments [13]. Consequently, a mainstay of therapy is the antiangiogenic small-molecule tyrosine kinase inhibitors (TKI) targeting the VEGF and platelet-derived growth factor β (PDGFβ) signaling pathways such as sunitinib, sorafenib, pazopanib and axitinib [14,15,16,17], the VEGF-targeted antibody bevacizumab [18] and the mammalian target of rapamycin (mTOR) inhibitors temsirolimus and everolimus [19]. Although these targeted agents demonstrate antitumor activity and prolonged progression-free survival (PFS), to date, metastatic RCC remains largely incurable [20]. Thus, the lack of long-term efficacy of current treatments reveals the urgent need for exploring mechanism-based therapies focusing on the different pathways involved in this devastating disease. Genomic analyses have recently identified the molecular mechanisms such as DNA damage response, intracellular signaling and immune engagement that may influence the response to cancer therapy [21]. Integration of genomic approaches with small-molecule and genetically based high-throughput screening holds the promise of improved discovery of candidate targets for cancer therapy [22,23]. To evaluate the functional assessment of drug–gene interactions, we developed a novel in-house multiplexed assay to study the cellular fitness of a panel of engineered ccRCC isogenic 786-O VHL^−^ cell lines in response to a collection of targeted cancer therapeutics including kinase inhibitors, allowing the interrogation of 2880 drug–gene pairs. This screening strategy revealed that renal cancer cells deficient in VHL were more vulnerable to the dual inhibition of the CK2 (casein kinase 2) and ATM (Ataxia telengestasia mutated) kinases than cells with wild-type VHL, establishing a lethal situation wherein both drug treatment and VHL deficiency led to lethality. ATM is a master controller of signaling networks, including DNA damage response (DDR), chromatin remodeling, cell growth, oxidative stress and senescence [24,25,26,27,28]. In addition, ATM signaling is involved in cancer, particularly in resistance to radio- and chemo-therapeutic treatment [29,30,31,32]. CK2 is a multifunctional protein kinase associated with a wide repertoire of substrates [33,34], which operates as a cancer driver by creating the cellular environment favorable to neoplasia [35]. Consequently, CK2 has emerged as a relevant therapeutic target being dysregulated in various cancers [36,37,38,39,40], including renal cancers [41,42]. 

Here, we demonstrated that inhibiting CK2 with the clinically relevant inhibitor CX-4945 in VHL-deficient renal cancer cells triggered a strong activation of phospho-ATM (Ser1982), suggesting that the benefit resulting from the combination of CK2 and ATM inhibition may be synergistic. Mechanistic investigations showed that the ATM inhibitor KU-60019 in combination with CX-4945 induced a strong inhibition of tumor cell proliferation, reduction of cell migration and reactive oxygen species (ROS)-dependent apoptosis in HIF-2α-expressing VHL-deficient cells. Using both monolayer cell cultures, tumor cells grown as spheroids as well as organotypic short-term cultures of renal tumor tissue slices, our findings reveal a selective killing of VHL-deficient renal carcinoma cells and provide a rationale for the use of ATM inhibitors in combination with CX-4945 in ccRCC.

## 2. Materials and Methods

### 2.1. Chemicals 

Lentiviral particles generated from the PLKO1 vector Hpgk-puro-cMV-tGFP containing different shRNA sequences targeting different genes were provided by Sigma-Aldrich (see Supplementary Table S1). The pGFP-C-shLenti containing ShNOX4 (NADPH Oxidase 4) from Origene was packaged in lentiviral particles.

All compounds were dissolved in dimethyl sulfoxide (DMSO) at a concentration of 10 mM. CX-4945 was synthesized at the Plateau Synthèse Organique, Département de Chimie Moléculaire, UJF, Grenoble, according to the method described in Reference [43]. The chemical library was composed of two commercial libraries, one from ENZO (screen-well kinases inhibitors) and the other from Selleckchem (Tyrosine kinase inhibitors), that were complemented with other inhibitors (see Supplementary Table S2). Carbobenzoxy-valyl-alanyl-aspartyl-[O-methyl]- fluoromethylketone (Z-VAD FMK) was from AdooQ biosciences, Propidium Iodide (PI), DMSO, poly(2-hydroxyethyl methacrylate), Tiron (4,5-dihydroxy-1,3-benzenedisulfonic acid) and Polybrene were from Sigma-Aldrich (Lyon, France), D-Luciferin Potassium salt was from Perkin Elmer (Every, France), Caspase 3/7 fluorescent reagent was from Essen Bioscience (Royston Hertfordshire, UK) and MitoSOX indicator and PrestoBlue™ Cell Viability Reagent were from Thermo Fisher Scientific (Villebon sur Yvette, France).

### 2.2. Cell Culture 

ccRCC cell lines 786-O and Caki-2 as well as normal renal proximal tubular epithelial cell line RPTEC (renal proximal tubular epithelial cell) were obtained from ATCC and Evercyte, respectively. The R305 cell line was a generous gift from Dr Arlot-Bonnemains. The 786-O cell lines were grown in 10 cm diameter plates in a humidified incubator (37 °C, 5% CO_2_) with RPMI 1640 medium (Gibco) containing 10% of fetal bovine calf serum, penicillin (100 U/mL) and streptomycin (100 μg/mL). RPTEC were cultured in ProXup (Evercyte). McCoy’s medium containing 10% of fetal bovine calf serum, penicillin (100 U/mL) and streptomycin (100 μg/mL) was used for the Caki-2 cell line. 786-O and RPTEC cells were passed every 2–3 days and only once a week, respectively. 

The parental VHL null cell line was used to generate its derivative lines containing either the empty expression vector HA-pBABE or a functional VHL construct HA-VHL (VHL^+^ cells). Stable transfectants were maintained in medium supplemented with 2 μg/mL puromycin. 

### 2.3. Transduction in 786-O Cells 

For infection, 786-O cells were plated into 6-well plates (8 × 10^5^) in 2 mL of serum-supplemented RPMI 1640 medium. The day after, adherent cells were incubated with lentiviral particles (1–5 MOI (multiplicity of infection)) diluted in 1 mL of serum-supplemented medium containing 8 μg/mL of polybrene. After 4 h, 1 mL of medium were added to cultures and transduction was maintained for 16 h before washing cells and changing the medium. For stable transduction, puromycin selection started 36 h post-infection (at the concentration of 2 μg/mL) and was maintained during all cell culture. 

### 2.4. Fully Automated, High-Throughput Screening Assay 

Control (Ctrl)- or targeted-shRNA expressing 786-O cells (90 μL, 55.5 × 10^3^ cells/mL) were seeded in 96-well plates and incubated overnight at 37 °C in a 5% CO_2_-humidified atmosphere. Then, 10 μL of each compound was added at the indicated concentrations (1, 5 or 20 μM final concentration) using the (Criblage pour des Molécules BioActives) CMBA’s robotic screening platform. DMSO was used as a negative control and 20 μM CX-4945 as a positive control. Plates were further cultured for 48 h. Cells were labeled with vital Hoechst (33342) together with Prestoblue and the fluorescence reading was made through the Tecan’s Infinite M100 reader. See the flow chart in Supplementary Figure S1. 

### 2.5. Data Processing and Hit Selection 

Primary and secondary high-throughput screening assays were evaluated and validated using a simple statistical parameter, the Z’-factor [44]. 

Raw data were first imported into the database of the homemade analysis software TAMIS, used for analysis. For each test plate and assay type (PrestoBlue or vital Hoechst), mean of the eight DMSO negative control values was considered as 0% activity and mean of the eight CX-4945 positive control values was considered as 100% activity. Then, each compound X-related raw value was translated in percent activity, compared to DMSO (0%) and CX-4945 (100%). Finally, for each compound X/targeted-shRNA Y pair and assay, the percentage of activity obtained with the compound X/Ctrl-shRNA was taken from the compound X/targeted-shRNA Y activity percentage in order to identify compound X/targeted-shRNA Y pairs showing additive, or even synergistic, effect compared to compound X/Ctrl-shRNA pairs. 

### 2.6. Guide RNA Design and Cloning

Briefly, the single-guide RNA (sg#1, sg#2 designed with https://zlab.bio/guide-design-resources/ and sgCTL) were cloned into the expression vector lentiCRISPRv2 backbone (addgene #52961) digested with BsmBI: A pair of 20 nt oligos containing the appropriate overhang was then ligated into the vector (Supplementary Table S3). After sequencing, lentiviral particles were produced to transduce 786-O cells according to Reference [45].

### 2.7. Two-Dimension (2D) Viability Assay 

Cytotoxicity was measured using PrestoBlue^®^ assay (Invitrogen, Carlsbad, CA, USA). Cell lines were seeded in a 96-well plate at a concentration of 5 × 10^4^ cells/mL. Cells were allowed to attach for 24 h at 37 °C and 5% CO_2_. The cells were exposed to the negative control DMSO or positive drug CX-4945 at 20 μM for 48 h or the other molecules at indicated concentrations. 

### 2.8. Western Blot Analysis 

Cells were lysed for 15 min in RIPA (Radioimmunoprecipitation assay) buffer (Tris HCl pH 7.4 10 mM, NaCl 150 mM, SDS (Sodium dodecyl sulfate) 0.1%, Na Deoxycholate 0.5%, EDTA (Ethylene diamine tetra acetic) 1 mM, Triton X100 1%, a protease inhibitor cocktail (Sigma P8340) and phosphatase inhibitor cocktails 1 and 2 (Sigma P2850, P5726)). Cells were subsequently clarified at 16,000 g for 15 min. Homogenate protein content was quantified using the BCA (bicinchoninic acid) protein Assay kit (Thermo Fisher Scientific). SDS-PAGE (Ploy acrylamide gel electrophoresis) was performed using pre-cast 4–12% gradient gel NuPAGE (Life technologies) and MOPS (3-(N-morpholino)propanesulfonic acid) running buffer at 150 volts for 75 min. Separated proteins (20 μg/lane) were transferred to PVDF membranes (60 min at 100 volts). Blotted membranes were blocked during 1 h at room temperature with saturation buffer (1% BSA in Tris-buffered saline containing 0.1% Tween 20), and then incubated with primary antibody diluted in saturation buffer for 16 h. Secondary antibodies were added for 1 h. Detection was performed by using Luminata Forte Western HRP (horse radish peroxidase) substrate (Millipore) and Fusion FX acquisition systems. GAPDH (Glyceraldehyde-3-phosphate dehydrogenase) or HSP90 (heat shock protein) were used to check for equal protein loading. Western blotting was performed using antibodies against human P-ATM Ser1981 #Ab81292, Glut1 #Ab15309, MDC1 #Ab11169, NOX4 #Ab109225 (Abcam, Paris, France), P-AKT Ser129 #AP3020a (Interchim, Montluçon France), GAPDH#AM4300 (Ambion, Villebon sur Yvette, France), HIF-2α #NB100122 (NovusBio, Cambridge, UK), ATM #2873, AKT #9272, PARP #9542, HSP90 #4874, purchased from Cell Signaling Technology (Ozyme, Saint-Cyr-L’École, France). The whole western blot figures can be found in the supplementary materials.

### 2.9. Three-Dimension (3D) Assay 

Cells were seeded into U-bottom 96-well plates coated with 20 mg/mL poly(2-hydroxyethyl methacrylate) (Sigma-Aldrich) at the density of 1500 cells per well and centrifuged at 400 g for 5 min. Three-day pre-formed spheroids were treated with the different drugs at indicated concentrations during 48 h. After that, Hoechst (200 ng/mL) and PI (0.5 μg/mL) were added as markers of cell nuclei and cell death, respectively. Images of PI (BGRFR_549_15 filter) and Hoechst (BGRFR_386_23 filter) fluorescence were acquired with a 5× objective on the ArrayScanVTI HCS microscope (Thermo Fisher Scientific, Villebon sur Yvette, France) at the CMBA platform. The HCS Studio Morphology Explorer bio-application was used to automatically perform High Content image Analysis and extract parameters such as spheroid area measurement, fluorescent intensity measurement of each staining and count of the dead cells number. 

### 2.10. Bulk RNA Barcoding (BRB)-Seq Library Preparation and Sequencing 

Total RNA was extracted from MCTS using the MirVana PARIS kit (Thermofisher). The 3′ Bulk RNA Barcoding and sequencing (BRB-seq) experiments were performed at the Research Institute for Environmental and Occupational Health (Irset, Rennes, France) according to the published protocol [46]. Briefly, the reverse transcription and the template switching reactions were performed using 4 µL total RNA at 2.5 ng/µL. RNA were first mixed with 1 µL barcoded oligo-dT (10 µM BU3 primers, Microsynth), 1 μL dNTP (desoxyribonucleoside triphosphate) (0.2 mM) in a PCR (Polymerase Chain Reaction) plate, incubated at 65 °C for 5 min and then put on ice. The first-strand synthesis reactions were performed in 10 µL total volume with 5 µL of RT (Reverse transcription) Buffer and 0.125 µL of Maxima H minus Reverse Transcriptase (Thermofisher Scientific, #EP0753) and 1 µL of 10 μM template switch oligo (TSO, IDT). The plates were then incubated at 42 °C for 90 min and then put on ice.

After reverse transcription (RT), decorated cDNA from multiple samples were pooled together and purified using the DNA Clean and concentrator-5 Kit (Zymo research, #D4014). After elution with 20 µL of nuclease-free water, the samples were incubated with 1 µL Exonuclease I (NEB, #M0293) and 2 µL of 10× reaction buffer at 37 °C for 30 min, followed by enzyme inactivation at 80 °C for 20 min.

Double-strand (ds) cDNAs were generated by PCR amplification in 50 µL total reaction volume using the Advantage 2 PCR Enzyme System (Clontech, #639206). PCR reaction was performed using 20 µL cDNA from the previous step, 5 µL of 10× Advantage 2 PCR buffer, 1 µL of dNTPs 50×, 1 µL of 10 µM LA-oligo (Microsynt), 1 µL of Advantage 2 Polymerase and 22 µL of nuclease-free water following the program (95 °C—1 min, 11 cycles: 95 °C—15 s, 65 °C—30 s, 68 °C—6 min, 72 °C—10 min). Full-length double-stranded cDNA was purified with 30 µL of AMPure XP magnetic beads (Beckman Coulter, #A63881), eluted in 12 µL of nuclease-free water and quantified using the dsDNA QuantiFluor Dye System (Promega, #E2670).

The sequencing libraries were built by tagmentation using 50 ng of ds cDNA with the Illumina Nextera XT Kit (Illumina, #FC-131-1024) following the manufacturer’s recommendations. The reaction was incubated for 5 min at 55 °C, immediately purified with DNA Clean and concentrator-5 Kit (Zymo research) and eluted with 21 µL of nuclease-free water. The tagmented library was PCR-amplified using 20 µL eluted cDNA, 2.5 µL of i7 Illumina Index, 2.5 µL of 5 µM P5-BRB primer (IDT) using the following program (72 °C—3 min, 98 °C—30 s, 13 cycles: 98 °C—10 s, 63 °C—30 s, 72 °C—5 min). The fragments ranging 300–800 base pairs (bp) were size-selected using SPRIselect (Beckman Coulter) (first round 0.65× beads, second 0.56×), with a final elution of 12 µL nuclease-free water. The resulting library was sequenced on an Illumina Hiseq 4000 sequencer as Paired-End 100 base reads following Illumina’s instructions. Image analysis and base calling were performed using RTA 2.7.7 and bcl2fastq 2.17.1.14. Adapter dimer reads were removed using DimerRemover (https://sourceforge.net/projects/dimerremover/).

### 2.11. BRB-Seq Raw Data Preprocessing

The first read contains 16 bases that must have a quality score higher than 10. The first 6 bp correspond to a unique sample-specific barcode and the following 10 bp to a unique molecular identifier (UMI). The second reads were aligned to the human reference transcriptome from the UCSC website (release hg38, downloaded in August 2020) using BWA version 0.7.4.4 with the non-default parameter “−l 24”. Reads mapping to several positions in the genome were filtered out from the analysis. The pipeline is described in Reference [47]. After quality control and data preprocessing, a gene count matrix was generated by counting the number of unique UMIs associated with each gene (lines) for each sample (columns). The resulting UMI matrix was further normalized by using the rlog transformation implemented in the DeSeq2 package [48]. Raw and preprocessed data were deposited at the ArrayExpress repository under the accession number E-MTAB-9977.

### 2.12. Bioinformatics Analysis

Principal component analysis (PCA) was performed using Python package sklearn v0.22. Histograms were generated using Python package seaborn v0.9.0. Dataframe manipulation and bar plot visualization were carried out using Python packages numpy v1.17.3 and pandas v0.25.3. Biological pathway enrichments were performed by Gene Set Enrichment Analysis (GSEA) (false discovery rate (FDR) < 0.25), using biological process (BP) and molecular function (MF) annotations from the GO.db_v3.10.0 (Bioconductor R3.6.3) database. ClusterProfiler v3.14.3 (Bioconductor R3.6.3) was used to execute GSEA and to generate visualizations using dotplot, emapplot and gsea plot functions.

### 2.13. 3D Cell Migration Assay 

The cell migration assay was performed as described in Reference [49]. Briefly, 3-day-old round-bottom plate pre-formed spheroids were transferred to flat-bottom 96-well plates and treated with either DMSO, KU-60019 (10 μM), CX-4945 (7.5 μM) or a mix of both for 48 h. Upon adherence to the solid support, spheroids disassembled and released cells migrated away radially from their initial position. Cell spreading was followed over 7 days. Markers, Hoechst 33,342 (blue) and PI (red), were added to visualize cell spreading and dead cells, respectively. Quantification was made using Image J software. 

### 2.14. ROS and Cell Death Measurements

Mitochondrial ROS production was measured with the MitoSOX indicator (5 µM), a fluorescent dye. Cell death was evaluated either with PI incorporation at 0.5 µg/mL or IncuCyte Caspase-3/7 reagents (5 µM). The IncuCyte^®^ (Sartorius, France) real-time imager performed image acquisitions every hour for 24/48 h. Images were analyzed for fluorescent area quantification with IncuCyte Zoom^®^ software (Royston Hertfordshire, UK).

### 2.15. Mice Orthotopic Tumor Xenograft Models

All animal studies were approved by the institutional guidelines and those formulated by the European Community for the Use of Experimental Animals. Renal orthotopic implantation was carried out by injection of 3 × 10^6^ 786-O luc cells into the left kidney of athymic nude mice as previously described [50]. Two patient derived xenograft (PDX) models, RCC43B and RCC-10-B, were generated by Xentech (Paris France). Briefly, tumor fragments (30 mm^3^) were grafted in the inter-scapular subcutaneous tissue, one fragment per mouse. Tumors were obtained from the lymph node’s metastatic sites of patients’ ongoing clear cell/granular kidney carcinoma. The status of the VHL gene was determined as wild type (WT) and mutated (E160fs14aa (chr3-10191481-G->GA-frameshift insertion)) for RCC43B and RCC10B, respectively.

### 2.16. Patients and Clinical Samples

All human renal carcinoma samples were obtained from patients, with their informed consent, and all procedures were approved by the ethics committee (Patient Protection Committee No. 2017 A0070251). Patients were recruited under the clinical trial, Comborein (NCT03572438). Fresh renal tumor tissue was obtained from patients undergoing a partial or a total nephrectomy for cancer at the Urology Department, University Hospital Center of Grenoble, Alpes (CHUGA).

### 2.17. Fresh Tissue Sectioning 

A Vibratome VT1200 (Leica Microsystems) was used to cut thin (300 μm) slices from fresh tumor tissue. Samples were soaked in ice-cold sterile-balanced salt solution (HBSS), orientated, mounted and immobilized using cyanoacrylate glue. Slicing speed was optimized according to tissue density and type; in general, slower slicing speed was used on the softer tissues and vice versa (0.03–0.08 mm/neoplastic tissue, 0.01–0.08 mm/s normal tissue). Vibration amplitude was set at 2.95–3.0 mm. 

### 2.18. Organotypic Tissue Cultures 

Tissue slices were cultured on organotypic inserts for up to 120 h (one slice per insert; Millipore). Organotypic inserts are Teflon membranes with 0.4 μm pores that allow preservation of 3D tissue structure in culture. Tissue culture was performed at 37 °C in a 5% CO_2_ humidified incubator using 2 mL of DMEM media supplemented with 10% inactivated fetal bovine serum (FBS) (GIBCO), 100 U/mL penicillin + streptomycin (Invitrogen) and placed in a rotor agitator to allow gas and fluid exchanges with the medium. The 786-Oluc tumor slices were harvested at baseline time (T0) and thereafter at 24 h intervals, and the slices were incubated with the drugs at the indicated concentrations and after 24 and 48 h and medium containing Luciferin was added and imaged using IVIS. Region of interest (ROI) levels allowed quantifying the efficacy of the drug treatment. The viability of PDX and human tumor slices was assayed as previously described [51].

### 2.19. Statistical Analysis 

The statistical significance of differences between the means of two groups was evaluated by using GraphPad version 6. Tests are indicated below in each figure legend.

## 3. Results

### 3.1. An Integrated RNAi and Drug Screen Identifies CK2 and ATM as Drug Targets in VHL-Deficient Renal Carcinoma Cells

Promising drug combinations may be discovered with synthetic lethal screens using siRNA or shRNA libraries to identify targets for anticancer drugs [52,53]. We performed a 786-O cell line-based synergy screen using custom-designed shRNA focused on genes involved in multiple hallmarks of cancer, including rapid proliferation, growth, survival and metastasis. The 786-O cells display a VHL frameshift deletion and a consequent increased HIF-2α and VEGF protein expression, making this cell line a workhorse for RCC research [54]. The collection of cell-silenced genes was designed in particular to include kinases that are required for cancer cell fitness (defined as genes required for cell growth or viability). These genes were selected according to their roles in different oncogenic pathways. This yielded a list of 36 genes that have been linked to several cancers, including ccRCC (Supplementary Table S1). To uncover combinatorial lethal interactions, these isogenic cell lines were individually screened against a custom small-molecule library. Among the 80 different targeted agents tested, 22 were Food and Drug Administration (FDA)-approved and used in the clinic, maximizing the chance of identifying drug–gene interactions that could be easily translated in a clinical setting. In particular, the library comprised clinically relevant kinase inhibitors (Supplementary Table S2). The drug library was screened at three concentrations, yielding 8640 data points. After 48 h of treatment, cell viability was determined with Hoechst (33,342) fluorescence and Prestoblue reading (Supplementary Figure S1). 

**Figure 1:**
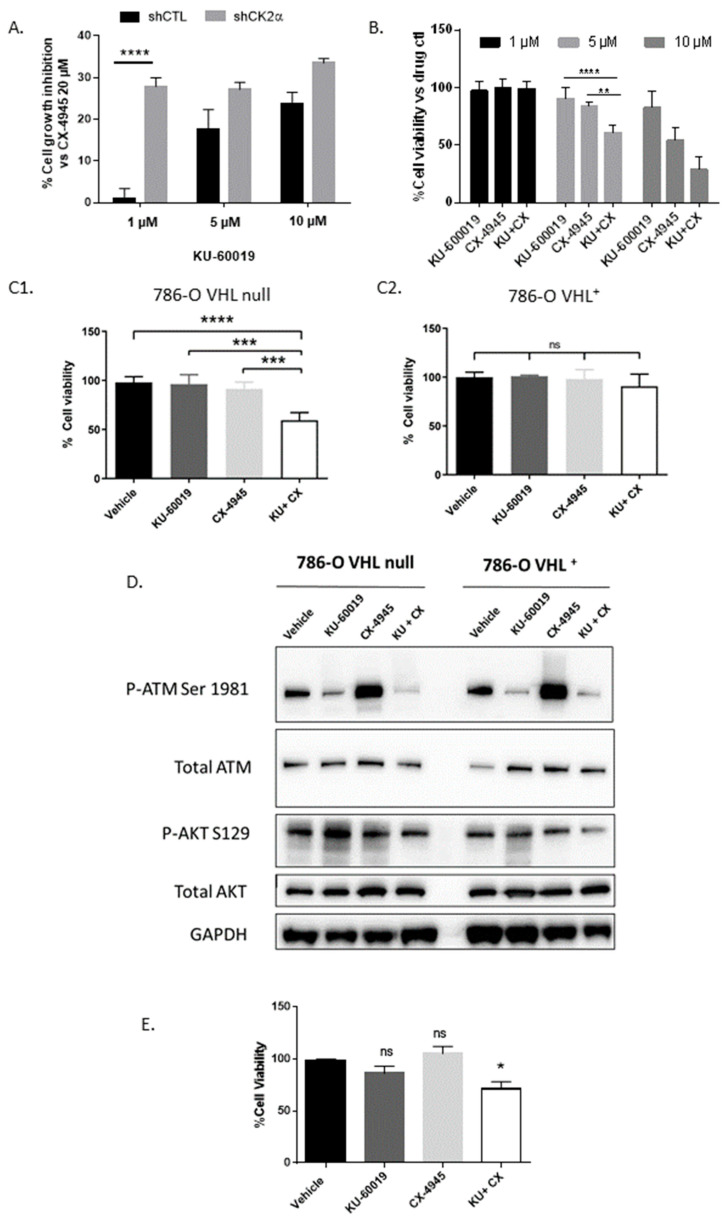
Co-Inhibition of CK2α and ATM kinases induces enhanced cell toxicity in ccRCC cells. (**A**) shCTL and shCK2α-transduced 786-O cells were treated with indicated concentrations of ATM inhibitor (KU-60019) for 48 h and cell viability was measured using Prestoblue^®^ assay (*n* = 4). The 100% cell growth inhibition corresponds to cells treated with CX-4945 (20 µM). (**B**) Relative sensitivity of 786-O cells to a dual drug inhibition of CK2α (CX-4945) and ATM (KU-60019) kinases at different concentrations (1 μM in black, 5 μM in light grey and 10 μM in dark grey) (*n* = 6). (**C**) Relative sensitivity of 786-O cells without VHL (**C1**, *n* = 4) or with re-introduced VHL (**C2**, *n* = 4) to single drugs (KU-60019, 5 µM, or CX-4945, 2.5 μM) or drug combination, in hypoxic conditions (1.5% O_2_). (**D**) Western blot analysis showing expression level of ATM, P-ATM (Ser1981), AKT, P-AKT (Ser129) and GAPDH as a loading control. (**E**) Relative sensitivity of CAKI-2 ccRCC cell line (*n* = 4) to single drugs (KU-60019, 5 µM, or CX-4945, 2.5 µM) or drug combination in hypoxic conditions (1.5% O_2_) compared to the Vehicle (DMSO). Two-way analysis of variance (ANOVA) and Mann–Whitney analysis were used for (**A**) and (**B**–**E**), respectively. * *p* < 0.05, ** *p* < 0.01, *** *p* < 0.001, **** *p* < 0.0001, ns (not significant).

The outcome of our combination-screening program revealed diverse patterns of drug sensitivities. From this pool, we identified a promising new combination between CK2α shRNA and the ATM kinase inhibitor KU-60019 that was prioritized for further evaluation (Figure 1A). Importantly, the potentiation of cell lethality was also observed when the cells were treated with a combination of KU-60019 and CX-4945, a specific CK2 inhibitor (Figure 1B). Since different VHL status in renal carcinoma cells have been shown to affect their drug sensitivity in hypoxic conditions [45,46], we stably reintroduced VHL into 786-O VHL-deficient cells and evaluated their sensitivity to the CK2 and ATM inhibitors under low oxygen concentration (1.5%) (Figure 1C1,C2). Under these conditions, both cell lines were weakly sensitive to each inhibitor alone. In contrast, we observed a strong inhibitory effect of their combination in the 786-O VHL^−^ cells compared to 786-O VHL^+^ cells. The combination led to a 37% cell death instead of a theoretical 10% cell death if considering a cumulative impact of the two inhibitors, suggesting a synergistic activity of this drug combination. Of note, this inhibitory effect was weaker in normoxic conditions, suggesting a role for the HIF signaling pathway in this response (Supplementary Figure S2). Western blot analysis was performed to visualize the targeted inhibition of ATM and CK2 in VHL^−^ and VHL^+^ 786-O cells under hypoxic conditions (Figure 1D). ATM was clearly inhibited by KU-60019, as evidenced by a reduction in the canonical ATM auto-phosphorylation at Ser1981 [55,56]. Likewise, CK2 was inhibited by CX-4945 as assessed by the reduction of P-AKT Ser 129 [57]. Both ATM and CK2 activities were strongly reduced by the combined drug treatment. Together, these observations demonstrate similar target engagement and biological activity in VHL^−^ and VHL^+^ 786-O cells. Surprisingly, CK2 inhibition led to a significant ATM activation, suggesting that combined inhibition of ATM and CK2 likely exerts its effect by affecting complementary signaling pathways that compromise cell viability. We next investigated whether the effect of this drug combination could also be observed in the Caki-2 cells, which express a mutated form of the VHL protein [58,59]. In hypoxic conditions, the viability of these cells was also altered by the drug combination (Figure 1E), whereas no significant effect was observed in RPTEC cells, which represent normal renal proximal tubule epithelial cells (Supplementary Figure S2C). Thus, our chemo-genetic screens revealed a vulnerability of VHL-deficient renal carcinoma cells to combined inhibition of CK2 and ATM kinases. Given the safety of CX-4945 in phase I clinical trials [60,61] and the lack of any reported connection between CK2 and ATM kinases, this drug combination was selected for further characterization and patented [62]. 

### 3.2. Combined Inhibition of CK2 and ATM Decreases Cell Migration and Promotes Apoptosis in Renal Multi-Cellular Tumor Spheroids

**Figure 2:**
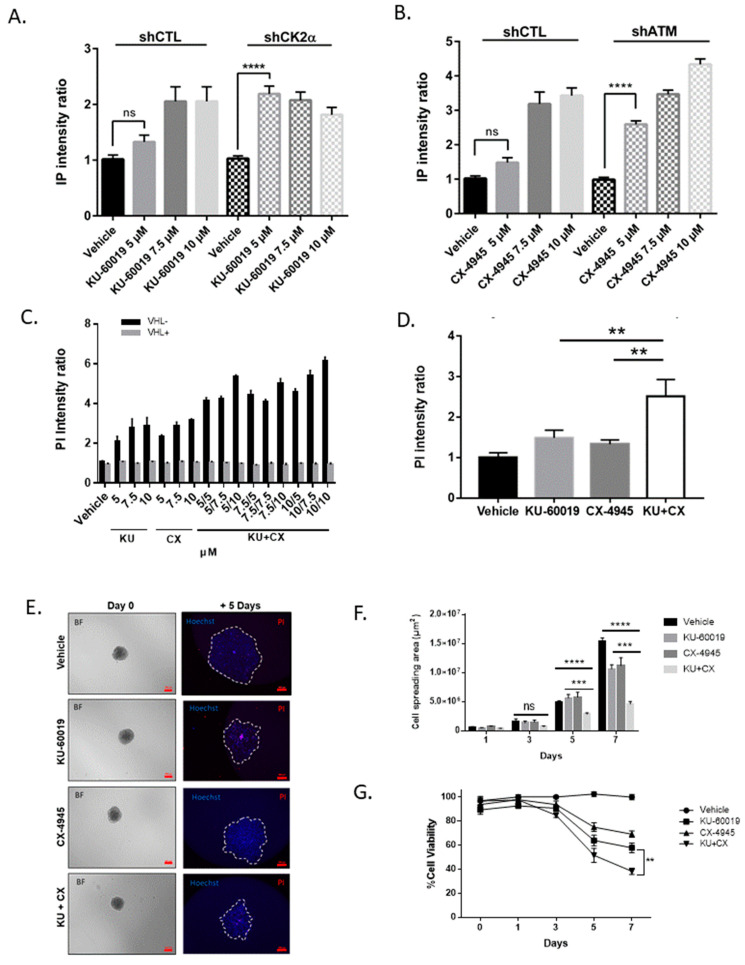
Cell death is induced in tumor environment conditions. (**A**,**B**) Multi cellular tumor spheroids (MCTS) were pre-formed for 3 days with indicated 786-O sh-transduced cell lines before treatment for 48 h with vehicle or increasing concentrations of KU-60019 or CX-4945 (5, 7.5 and 10 μM). Cell death was monitored by PI quantification using the ArrayScan^®^ VTI HCS Reader (Thermo Fisher Scientific, Villebon sur Yvette, France). A significant difference (**** *p* ≤ 0.0001) was observed when comparing the treatment of either shCK2α MCTS to Vehicle (DMSO) with 5 μM KU-60019 (**A**) or shATM MCTS to vehicle with 5 μM CX-4945 (**B**) (Kruskal–Wallis non-parametric test, *n* = 20). (**C**) MCTS of 786-O VHL^−^ (*n* = 19, black bars) or 786-O VHL^+^ (*n* = 12, grey bars) cells were treated with increasing concentrations of KU-60019 and/or CX-4945 (5, 7.5 or 10 µM each) or Vehicle (DMSO). PI (0.5 µg/mL) incorporation was quantified at 48 h using the ArrayScan^®^ VTI HCS Reader. (**D**) R305-derived MCTS were treated with either vehicle (DMSO), drugs alone (KU-60019 10 μM; CX-4945 5 μM) or in combination (KU + CX) for 48 h before cell death quantification (Kruskal–Wallis non-parametric test, *n* > 12). (**E**) Representative pictures of spheroids that were placed on plastic for migration assay after day 0 and day 5 of treatment (Scale bar at day 0:100 μm, Scale bar at day 5:500 μm). (**F**) Kinetic analysis of cell spreading. (**G**) Cell viability kinetics after 0, 1, 3, 5 and 7 days of cell spreading (Kruskal–Wallis test, *n* = 15). ** *p* < 0.01, *** *p* < 0.001, ns (not significant).

In order to better simulate the tumor environment, drug treatments were performed on multi-cellular tumor spheroids (MCTS), which are known to mimic micro-tumors more closely than cancer cell line monolayers. In addition, several studies reported that drug sensitivity testing performed on MCTS can efficiently predict the efficacy of new antitumor compounds [63,64]. Therefore, MCTS generated from shCK2α-786-O or shATM-786-O cells were treated for 48 h with increasing concentrations of KU-60019 or CX-4945 respectively, and cell death was monitored by PI quantification. As shown in Figure 2A, B, significant cell death was specifically induced at the lowest concentrations (5 µM) of KU-60019 or CX-4945 in shCK2α- or shATM-786-O VHL-deficient cells, respectively. Furthermore, cell death induction was also observed in MCTS generated from parental VHL^−^ 786-O cells treated with KU-60019, CX-4945 alone or in combination (Figure 2C). In contrast, VHL^+^ 786-O MCTS were insensitive to the drug combination at any concentration. Similar results were observed with another ccRCC VHL^−^ patient-derived cell line (R305) (Figure 2D) [65]. 

Migration is one of the first mechanisms that cancer cells use to escape from a primary tumor before metastatic colony formation in distant organs [65]. Cell spreading from MCTS might predict cell migration [49]. Thus, we determined the capacity of 786-O cells to migrate and escape from MCTS and to survive after 1 to 7 days of treatment with the drugs alone or in combination. As shown in Figure 2E,F, after 5 days of culture, there was a statistically significant decrease in cell spreading after CX-4945/KU-60019 combination regimen, as compared with single treatments, indicating a synergistic effect of this drug combination. Moreover, the viability of migrating cells was much more strongly affected by the combination than by the single drugs (Figure 2G). 

**Figure 3:**
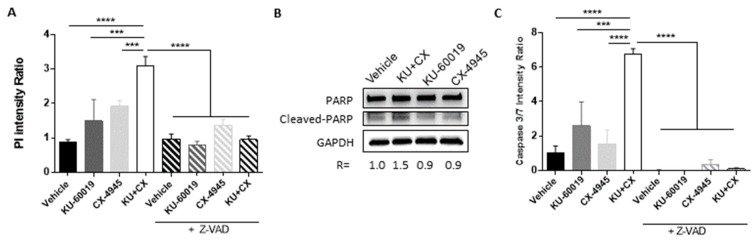
Combined CK2/ATM inhibition induces apoptosis in MCTS. (**A**) 786-O VHL^−^ MCTS were incubated with PI (0.5 µg/mL) and the following treatments: Vehicle (DMSO), KU-60019 (10 µM) and/or CX-4945 (5 µM) ± Z-VAD (15 µM). Cell mortality (PI incorporation) was assessed over 48 h using IncucyteZOOM. PI incorporation under treatments is represented at 48 h (*n* = 10). (**B**) Western blot analysis after a 24 h treatment showing expression level of PARP, cleaved-PARP and GAPDH as loading control. (**C**) 786-O VHL^−^ MCTS were incubated with Caspase 3/7 Fluorescent reagent (5 µM) with the indicated treatments as in (**A**). Apoptosis was assessed over 24 h using IncucyteZOOM. Cleaved-caspase detection is represented after 16 h of treatment (*n* = 6). One-way ANOVA statistical analysis was used, *** *p* < 0.001, **** *p* < 0.0001.

To further evaluate whether the increase of PI staining observed after drug treatment was related to apoptosis, VHL-786-O MCTS were treated with KU-60019 or CX-4945 alone or in combination, in the absence or presence of the pan-caspase inhibitor Z-VAD. After 48 h of treatment, cell death either induced by the drugs alone or by their combination was abrogated by the presence of Z-VAD, suggesting that the drug combination cooperatively induced apoptosis in 786-O MCTS (Figure 3A). In agreement with these results, enhanced poly (ADP-ribose) polymerase (PARP) cleavage was observed in response to the drug combination (Figure 3B). Similarly, IncuCyte^®^ real-time imaging showed that the presence of Z-VAD completely thwarted the strong Caspase 3/7 activation observed in response to CX-4945/KU-60019 treatment (Figure 3C). Therefore, these results indicate that apoptosis induction by the CX-4945/KU-60019 combination strongly compromises the viability of 786-O cells inside the MCTS. 

### 3.3. HIF-2α Expression Enhances Vulnerability to Combined CK2 and ATM Inhibition

**Figure 4:**
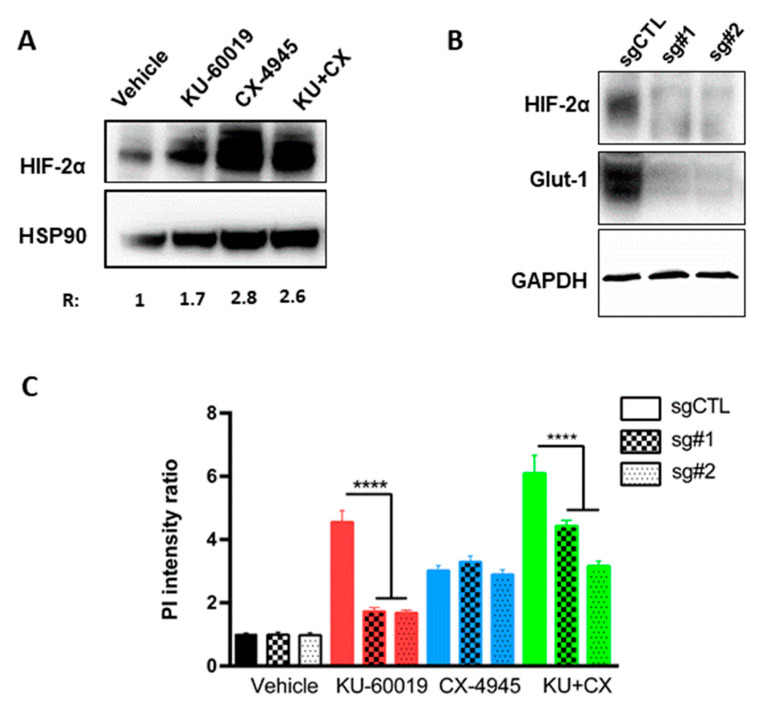
HIF-2α expression enhances vulnerability to the CX-4945/KU-60019 combination. (**A**) 786-O VHL null MCTS were treated either with vehicle (DMSO), KU-60019 (10 μM) and/or CX-4945 (5 μM) for 48 h. Western blot analysis shows protein expression levels of HIF-2α and HSP90 as loading control. (**B**) Protein extracts from HIF2-α CRISPR/CAS9 (sg#1 and sg#2) and CTL CRISPR/cas9 (sgCTL) 786-O cells culture in 2D were analyzed by Western blot. Shown are expression levels of HIF2-α, Glut-1 and GAPDH as a loading control. (**C**) MCTS, as described in (**B**), were treated either with vehicle (DMSO), KU-60019 (10 μM) and/or CX-4945 (5 μM) for 48 h. Cell death was quantified by PI measurement (*n* = 9). One-way ANOVA statistical analysis was used, **** *p* < 0.0001.

Since the 786-O VHL^−^ cell line lacks wild-type HIF-1α but expresses HIF-2α [66], we investigated the effect of the drugs alone or in combination on HIF-2α expression (Figure 4A). After 48 h of treatment, KU-60019 or CX-4945 alone or in combination led to significantly increased HIF-2α expression. Conversely, we wondered whether the sensitivity of 786-O VHL^−^ cells to combined CX-4945/KU-60019 treatment was affected by loss of HIF-2α. For this, 786-O VHL^−^ cells were transfected with Clustered Regularly Interspaced Short Palindromic Repeats (CRISPR)/CAS9 plasmids to generate HIF-2α knockout cell lines in which HIF-2α and its downstream target Glut-1 were downregulated (Figure 4B). MCTS generated from these cells were treated for 48 h with KU-60019 or CX-4945 alone or in combination and cell death was quantified by PI measurement. Strikingly, as shown in Figure 4C, the capacity of KU-60019 alone or in combination with CX-4945 to induce cell death in these MCTS was impeded in an on-target manner by CAS9-mediated loss of HIF-2α, suggesting that the vulnerability to combined inhibition of ATM and CK2 in VHL-deficient ccRCC is positively correlated with HIF-2α expression levels. Thus, HIF-2α acts as a mediator that potentiates CK2 and ATM inhibition to induce cytotoxicity in VHL-deficient renal carcinoma cells. Therefore, the level of HIF-2α expression may represent a potential efficacy biomarker for the CX-4945/KU-60019 treatment (a higher baseline pre-treatment level of HIF-2α would be predictive of response for patients with ccRCC). 

Phosphorylation of HIF-1α and HIF-2α subunits have been demonstrated to enhance transactivation of target genes by either disrupting HIFα interaction with VHL and thereby stabilizing HIFα, or by increasing the affinity of HIFα for transcriptional coactivators [67]. CK2 was described as a regulator of HIF-1α transcriptional activity [68] and hypoxia-induced phosphorylation by CK2 has been demonstrated in the C-TAD domain at conserved threonine residues (Thr796 for HIF-1α and Thr840 for HIF-2α). Mutation of these residues decreased reporter activity [69,70,71]. In contrast, the involvement of CK2 in the regulation of HIF-2α has not been established. To assess a potential link between CK2 and HIF-2α, we first analyzed, by ion-exchange chromatography, cell extracts from HEK293T cells expressing HA-tagged HIF-2α. Both CK2 activity assays and Western blot analysis of CK2α/α’ subunits revealed co-elution of CK2 with HIF-2α (Supplementary Figure S3A). Fractions containing the highest CK2α/α’ levels (fractions 9–12) were incubated with [γ-^32^P]-ATP/MgCl_2_ in the absence (DMSO) or presence of CX-4945 and phosphorylated proteins were resolved on SDS-PAGE and detected by autoradiography. A phosphorylated protein with the expected size of HIF-2α (110 kDa) was co-eluting with CK2α/α’ and this phosphorylation was abolished in the presence of CX-4945 (Supplementary Figure S3B). Alternatively, HA-HIF-2α was immunoprecipitated from transiently transfected HEK293T cells and the immune complexes were either incubated with [γ-^32^P]-ATP/MgCl_2_ (Supplementary Figure S3C-a) or assayed for CK2 kinase activity (Supplementary Figure S3C-b). A co-immunoprecipitated kinase activity was able to phosphorylate a protein with the expected size of HIF-2α and this phosphorylation was abrogated in the presence of CX-4945 (Supplementary Figure S3C-a). Likewise, HA-HIF-2α immunoprecipitates were shown to contain a CX-4945-sensitive CK2 activity (Supplementary Figure S3C-b). Thus, HIF-2α and CK2 co-purify as a stable complex throughout ion-exchange chromatography or after immunoprecipitation of HIF-2α. 

Collectively, these experiments suggest that HIF-2α-dependent vulnerability to combined inhibition of ATM and CK2 in VHL-deficient renal carcinoma cells may rely on a functional CK2–HIF-2α interaction. 

### 3.4. Cell Death in Response to Combined CK2 and ATM Inhibition Is Dependent on ROS Overproduction

**Figure 5:**
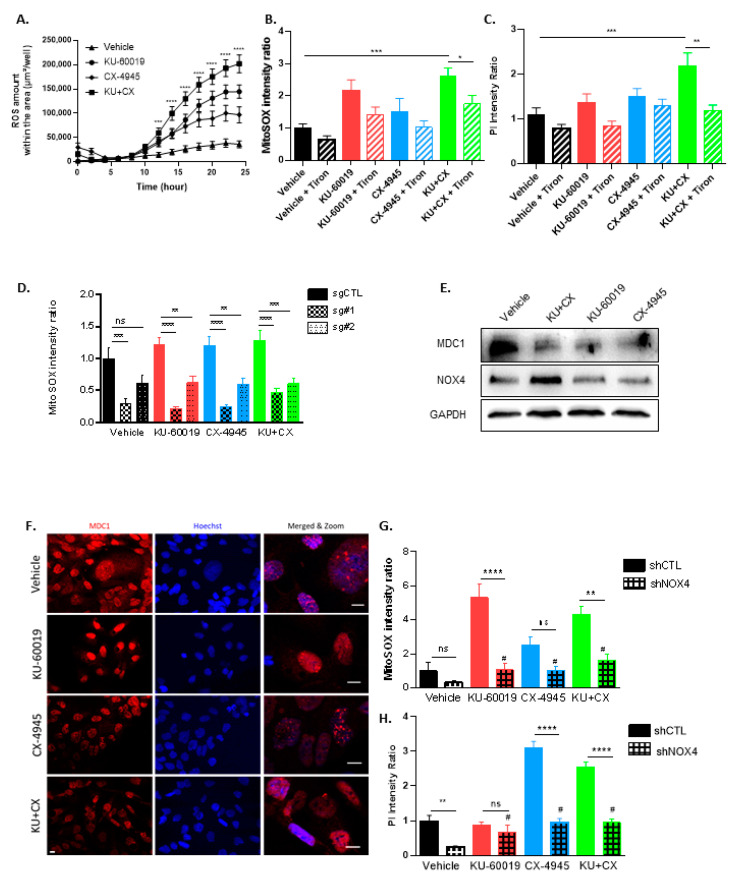
ROS-mediated apoptosis induced by CK2 and ATM co-inhibition. (**A**–**C**,**E**) MCTS of 786-O VHL^−^ cells treated with Vehicle (DMSO, 0.1%), KU-60019 (10 µM) and/or CX-4945 (5 µM) ± Tiron (400 µM) for 24 h (**A**,**B**) or 48 h (**C**). (**D**) MCTS of 786-O HIF-2α CRISPR/CAS9 (Sg#1 and Sg#2) and 786-O CTL CRISPR/CAS9 (sgCTL) were treated either with vehicle (DMSO) or KU-60019 (10 μM) and/or CX-4945 (5 μM) for 48 h. (**F**) Fluorescence images of 786-O VHL^−^ cells stained with MDC1 and Hoechst after a 48 h treatment with DMSO (control, 0.1%), KU (5 µM) and/or CX (2.5 µM), scale bar = 20 µm. (**G**,**H**) MCTS of 786-O VHL^−^ shNOX4D and 786-O VHL^−^ shCTL cells treated with Vehicle (DMSO, 0.1%), KU (10 µM) and/or CX (5 µM) for 24 h (**G**) or 48 h (**H**). (**A**,**B**,**D**,**G**) ROS production (O2•^−^) was assessed over 24 h using the MitoSOX fluorescent reagent (5 µM) in real-time using the IncucyteZOOM Imager. ROS production under treatments is represented over 24 h in (**A**) (*n* = 36), while (**B**) (*n* = 12), (**D**) (*n* = 9) and (**G**) (*n* = 12) represent the ROS production at 18 h. (**C**,**H**) MCTS were treated simultaneously with PI (0.5 µg/mL) and drugs. Cell mortality (PI incorporation) was assessed over 48 h using IncucyteZOOM. PI incorporation under treatments is represented at 32 h (**C**, *n* = 24) and 48 h (**H**, *n* = 24). (**E**) 786-O VHL^−^ MTS Western blot analysis showing protein expression levels of MDC1, NOX4 and GAPDH as loading control. Two-way ANOVA and one-way ANOVA were used for kinetics and plots, respectively. * *p* < 0.05, ** *p* < 0.01, *** *p* < 0.001, **** *p* < 0.0001, ns: not significant, #: not significant compare to Vehicle-shCTL.

Oxidative stress, defined as a relative excess of ROS, has been implicated in many aspects of cancer biology. While ROS might be pro-tumorigenic, high ROS levels are cytotoxic [72]. Owing to their active metabolism and oncogenic stimulation, most cancer cells exhibit elevated levels of ROS [73] that make them more vulnerable than normal cells to additional oxidative stress [74,75,76]. Therefore, an emerging view is that such vulnerability can be exploited to selectively kill cancer cells [77]. As mitochondria are known to play a central role to elicit apoptosis in response to many stresses [78,79], we investigated whether mitochondria are involved in KU-60019/CX-4945-induced apoptosis. Mitochondrial ROS generation was analyzed using the MitoSOX Red mitochondrial superoxide indicator. We found that the KU-60019/CX-4945 combination triggers a much stronger and more sustained ROS generation than the drugs alone in VHL^−^ 786-O MCTS. This effect was evident as early as 12 h post-treatment (Figure 5A and Supplementary Figure S4A,B). Results in Figure 5B showed that Tiron, a cell-permeable ROS scavenger, mitigated this robust ROS overproduction. To interrogate the causal relationship between ROS increase and cell death upon combined treatment, we hypothesized that counteracting ROS generation with Tiron would prevent apoptosis. Apoptosis was significantly detectable after 48 h. Tiron, by blunting ROS, was effective in preventing the apoptosis induced by the drug combination (Figure 5C). These results strongly suggest that mitochondrial ROS overproduction is critical in KU-60019/CX4945-induced apoptosis. 

In hypoxic conditions, HIF-1α is activated by ROS and regulates the redox status of cells [80]. As HIF-2α expression enhances vulnerability to combined inhibition of ATM and CK2 (Figure 4C), we examined the mitochondrial ROS accumulation in HIF-2α-depleted 786-O cells. We found that in both control- and drug-treated cells, ROS production was downregulated in the absence of HIF-2α (Figure 5D). These results are in accordance with the reduced drug-induced apoptosis in HIF-2α-depleted cells (Figure 4C). 

ROS are highly active oxygen-containing molecules that can induce DNA double-strand breaks (DSBs) [81]. The cellular response to DSBs involves ATM, which phosphorylates essential components of a macromolecular complex, including the Mediator of DNA damage Checkpoint 1 (MDC1) [82,83]. To understand the link between ATM and the oxidative stress, we examined the effect of the drug treatment on the expression and localization of the MDC1 protein in 786-O MCTS. MDC1 expression was diminished and its localization in nuclear foci was lost upon MCTS drug treatments with either KU-60019 alone or in association with CX-64945 (Figure 5E,F). NADPH oxidases (NOXs) are essential membrane-bound enzyme complexes for ROS production [84] and high-level expression of the NOX4 isoform was observed in the kidney [85], where it plays a regulatory role in intracellular redox homeostasis [86]. Thus, the role of NOX4 in ROS generation was analyzed in 786-O cells upon exposure to the drug combination. As shown in Figure 5E, NOX4 expression level increased in response to the combination treatment. NOX4 was then knocked down in 786-O cells to evaluate its role in mitochondrial ROS production and cell death (Supplementary Figure S5). Indeed, ROS production was significantly decreased in shRNA-NOX4 cells upon KU-60019 or KU-60019/CX-4945 combination treatments as compared to shRNA-CTL cells (Figure 5G). Likewise, PI incorporation in response to drug treatments was strongly reduced in NOX4-knockdown 786-O cells treated with CX-4945 or the KU-60019/CX-4945 combination (Figure 5H).

**Figure 6:**
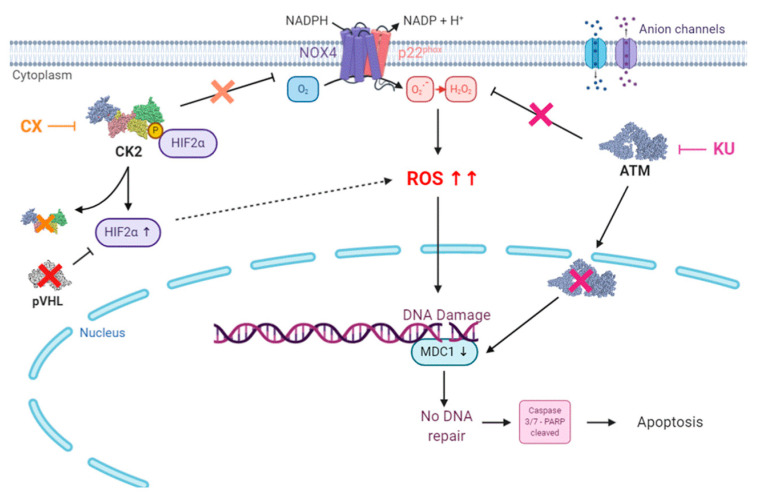
Proposed mechanistic model for the combinational drug therapy targeting CK2 and ATM: In renal cells, the NADPH oxidase 4 (NOX4) enzyme is responsible for ROS production. NOX4 activity is downregulated by CK2-dependent phosphorylation. Inhibition of CK2 by the CX-4945 inhibitor releases the CK2-dependent NOX4 inhibition, leading to ROS generation and HIF-2α induction. ATM inhibition by KU-60019 also induces ROS generation. ROS induced DNA damages and ion channels expression alteration. Both ATM and CK2 are involved in DNA repair machinery. Therefore, their inhibition prevents ROS-induced DNA damages repair, leading to apoptotic cell death. Loss of functional VHL leads to HIF-2α accumulation. HIF-2α appears as a biomarker of which expression level is correlated with sensitivity to this combined CK2/ATM inhibition (Direct link, full line; indirect link, dashed line).

Mechanistically, these results suggest that the combined inhibition of CK2 and ATM in renal cancer cells triggers an HIF-2α/NOX4-dependent ROS overproduction, leading to cellular damages and ultimately to Caspase-mediated irreversible cell death (Figure 6).

### 3.5. KU-60019/CX-4945 Combination Leads to a Stronger Transcriptome Deregulation Than the Drugs Alone

Outcomes of excessive levels of ROS include altered cell signaling, impaired energy metabolism and cell transport mechanisms [87]. Gene expression-based pathway analysis confirmed the expected positive association between ROS overproduction and alteration of various ion channel functions [88]. To explore the overall impact of our drug combination on renal tumor cell biology, we performed transcriptome profiling of MCTS generated from 786-O cells treated with vehicle only (DMSO), KU-60019, CX-4945 or their combination. As we performed only one biological replicate per experimental condition for this first exploratory analysis, we focused on major changes in biological processes. Sequencing of the transcriptomes by BRB-seq and their filtering by removal of genes with null expression variance or quantified with a low number of reads allowed to estimate the expression of 5866 genes (Supplementary Table S3A). Classification of the transcriptomes by principal component analysis (PCA) showed that MCTS treated with CX-4945 or the KU-60019/CX-4945 combination were clearly separated from the control MCTS treated with DMSO (Supplementary Figure S6A). On the other hand, the transcriptome for the KU-60019 condition was much closer to the control, suggesting a weaker impact of KU-60019 on the overall deregulation of the MCTS transcriptome. Detection of genes deregulated for each treatment relative to the DMSO condition (Log2(gene expression fold change) > 0.3) showed a greater number of deregulated genes for KU-60019/CX-4945 (271 genes) compared to KU-60019 (193 genes) or CX-4945 (254 genes) (Supplementary Figures S6B and S7A,B). Identification of significantly enriched ontological terms was carried out with the Gene Set Enrichment Analysis (GSEA) method and Gene Ontology (GO) resources (Supplementary Table S3). For this analysis, we used the Log2 (gene expression fold change) calculated between treatments for each of the 5866 expressed genes. A greater number of biological processes (BP) and molecular functions (MF) were identified as significantly enriched following treatment with KU-60019/CX-4945, compared to CX-4945 alone. No ontological term was found enriched after KU treatment (Supplementary Figure S6C). Then, we generated an enrichment map to get a global view of BP terms found significantly enriched by GSEA following KU-60019/CX-4945 treatment (Supplementary Figure S6D). This visualization, which clusters together sets of mutually overlapping genes, identified two major functional modules: one module, repressed by KU-60019/CX-4945, associated with mitochondrial energy production and metabolism, and another module, activated by the combination, associated with the cell cycle. Interestingly, the activated processes for this cell cycle-related module highlighted the negative regulation of nuclear division and chromosome segregation, suggesting a downregulation of the cell cycle by the over-expression of these sets of genes. The same analysis, using BP terms enriched following treatment with CX-4945 alone, showed the presence of the same two major functional modules (Supplementary Figure S8A). In agreement, CK2 inhibition has already been shown to affect the cell cycle, metabolism and mitochondrial functions [40,89,90,91]. Furthermore, a comparison of the levels of deregulation of the major biological processes, defined by the Normalized Enriched Score (NES), showed that they were overall more deregulated in the KU-60019/CX-4945 condition than with CX-4945 alone (Supplementary Figure S6E). Of note, the “apoptotic DNA fragmentation” process was found among the BP terms specifically enriched following combined treatment (Supplementary Figure S6F). Regarding the MF terms enriched by GSEA, we found that two major repressed functional modules were shared by KU-60019/CX-4945 and CX-4945 treatments: a module associated with oxidation-reduction and another module involved in ionic transport (Supplementary Figures S7C and S8B) [92]. Again, the deregulation levels of these shared MF terms showed that they tended to be more repressed by the KU-60019/CX-4945 combination than with CX-4945 alone (Supplementary Figure S7D). Therefore, this first cartography shows that the stronger transcriptome deregulation in response to KU-60019/CX-4945 compared to drugs alone may compromise cell viability in MCTS.

### 3.6. Cooperative Blockade of CK2 and ATM Induces Cell Death in Ex Vivo Human Renal Tumor Slice Cultures 

**Figure 7:**
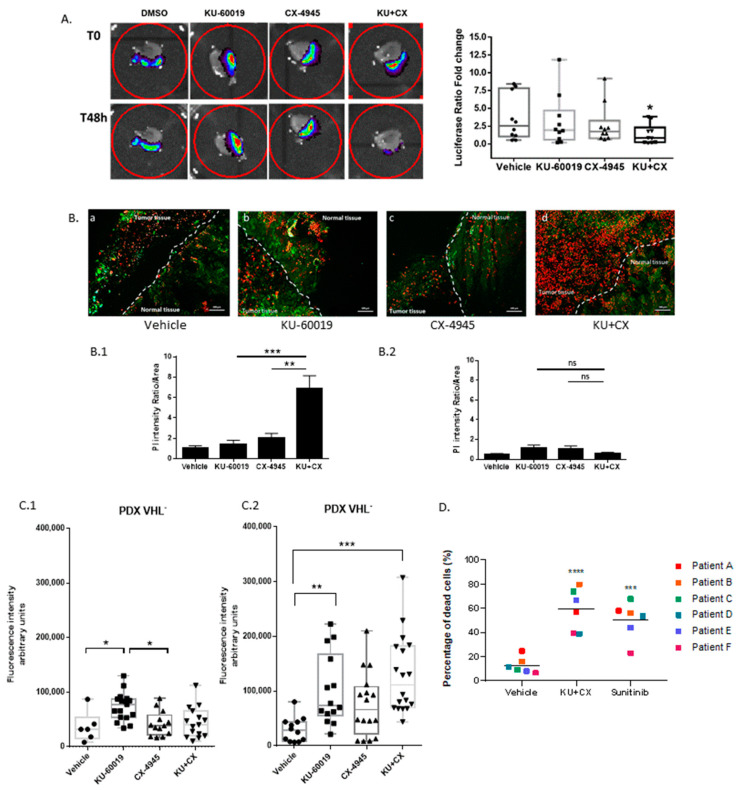
Ex vivo models as pre-clinical tests for drug combination efficacy. (**A**) Tissue slices derived from 786-O-Luc xenografts were imaged for Luciferase activity before treatment and 48 h after incubation with indicated molecules (dots: vehicle, square: 10 μM ATM triangle: 10 μM CX-4945, inverse triangle: 10 μM ATM^+^ 10 μM CX-4945). Right panel: Luciferase ratio fold changes (Treated versus Control) after 48 h of treatments (* *p* ˂ 0.05). Mann–Whitney test, *n* = 10. (**B**) Tissue slice cultures carrying tumor and normal kidney tissue (indicated in the pictures) were labeled with Live and Dead Kit after 48 h of treatments with (**a**) Vehicle, (**b**) KU-60019 10 μM, (**c**) CX-4945 10 μM and (**d**) KU 10 μM + CX 10 μM. Intensity of PI (Red marker = dead cells) was measured in both normal and tumor areas. (**B.1**) Significant difference was observed between KU-60019 (*** *p* = 0.0005), CX-4945 (** *p* = 0.007) alone and KU + CX, *n* > 20. (**B.2**) PI intensity in normal tissue shows no significant difference when comparing each drug alone versus drug combination. Scale bar 100 μm (Mann–Whitney test for all describe conditions). (**C**) Tissue slice cultures of two PDX RCC (patient derives xenograft renal cell carcinoma) models either VHL-null (**C.1**) or VHL-positive (**C.2**) were treated and analyzed as in (**B**) (Nonparametric, one-way ANOVA test, * *p* < 0.05, ** *p* < 0.01, *** *p* < 0.001, *n* ≥ 6). (**D**) Tissue slice cultures of patients’ tumors were recovered from the hospital (COMBOREIN Clinical Trial NCT03571438), treated for 48 h with sunitinib 10 µM or as in (**B**) and stained with Live and Dead Kit. Significant difference observed between vehicle and treatments, one-way ANOVA, *** *p* < 0.001, **** *p* < 0.0001, *n* = 6, ns not significant.

Patient-derived tumor xenografts and tumor organoids have become important preclinical model systems for cancer research. However, appropriate stromal and competent immune environments are lacking in both model systems [93]. Monolayer cell cultures as well as MCTS also have inherent limitations in evaluating the role of the tumor microenvironment in the response to therapies and their actual efficacy in humans. Ex vivo tissue slices that maintain cellular architecture, while also preserving the integrity of the tumor–stroma interaction, are promising models [94]. This method, which takes advantage of the rapid sectioning of tumors immediately after harvesting, allows for the investigation of antitumor pharmacological properties in a system that preserves the original cancer microenvironment for up to 5 days ex vivo [95]. Our previous study established a proof of principle of tumor tissue slice cultures for the evaluation of molecularly targeted therapies in renal cancer [51]. We applied this methodology to three different models: (1) tissue slices generated from Luc-expressing 786-O tumor xenografts, (2) patient-derived tumor xenografts (PDX) and (3) human ccRCC tumors obtained immediately after surgical resection. As shown in Figure 7A, after 48 h treatment of tumor slices derived from 786-O-Luc xenografts, luciferase luminescence was barely affected by KU-60019 or CX-4945 alone, whereas the luminescence signal was significantly inhibited by the drug combination. Viability assays also revealed that the drug combination exhibited greater efficacy than a single agent to induce cell death in tumor tissue slices (Figure 7B(B.1)). Importantly, cell viability of normal tissue was essentially unaffected by the drug combination, indicating its low toxicity to normal tissue/cells of the xenograft slices (Figure 7B(B.2)). PDX models more accurately recapitulate the clinical trial situation and it has been shown that RCC tumors are well-suited for the development of tumor-graft models in which tumors derived from patients are implanted in mice [96]. Therefore, the effect of ATM and CK2 inhibition was evaluated on ex vivo tumor slice cultures derived from two VHL^−^ and VHL^+^ ccRCC PDX models, respectively. As illustrated in Figure 7C.1,C.2, KU-60019 induced a significant cell death in both models, whereas the combination was only effective in the VHL^−^ tumor samples (Figure 7C.2). To get even closer to a preclinical patient-relevant setting for predicting patient response to drugs, the effect of the KU-60019/CX-4945 combination was compared to the current standard of care sunitinib treatment in tumor tissue slices derived from six ccRCC patients (Figure 7D). Although patient tissue slices responded to sunitinib with increased cell death, the KU-60019/CX-4945 combination was more efficient than sunitinib in all patient tissue slices except for patient D. 

Altogether, these data illustrate the therapeutic predictive value of the tumor tissue slice models and demonstrate the significant therapeutic potential of the KU-60019/CX-4945 combination in patients with ccRCC. 

## 4. Discussion

The most common type of renal cell carcinoma (ccRCC) is characterized by inactivation of VHL leading to HIF stabilization and increased transcription of HIF target genes. Consequently, since 2005, nine agents targeting the VHL-HIF pathway have been approved by the FDA for the treatment of patients with advanced kidney cancer. For example, temsirolimus, which inhibits HIF through the targeting of mTOR, and antiangiogenic agents such as sunitinib or sorafenib have shown some effectiveness in the management of RCC [19]. However, the potential clinical benefits of these molecules are tempered because tumors ultimately progress regardless of these therapies and few patients are cured of this disease [97], thus calling for combined intervention on complementary pathways that can engender drug resistance to individual targeted drugs. In this vein, we previously showed that combined inhibition of phosphatidylinositol 3-kinase (PI3K) and Src kinases exhibits synergistic therapeutic efficacy in clear-cell renal carcinoma [50]. 

Here, we combined genetic and chemical screens to systematically search for lethal interaction in VHL-deficient renal cancer cells. This multiplexed assay allowed the interrogation of thousands of gene–drug combinations with the potential to identify clinically relevant interactions that could lead to new patient-stratified treatment. Among the effective combinations identified from this screen, we found that a novel combination, pairing the CK2 inhibitor CX-4945 with the ATM inhibitor KU-60019, dramatically enhanced cell death in VHL-deficient renal carcinoma cells. CK2 has effects on a broad range of cellular functions participating to the (DDR) [98] and to the regulation of chromatin modifiers [99,100,101,102,103]. More specifically, its implication in tumor development and recurrence may be related to its double-edge functions in promoting cell growth and inhibiting apoptotic cell death [104] and in the development of multidrug resistance phenotypes [35,57]. Modest alterations in the levels of CK2α are sufficient to induce dramatic effects on cell fate, resulting in potent induction of apoptosis [105]. We previously showed that CK2α is overexpressed in renal cancers, suggesting its potential relevance as a therapeutic target [41]. Consequently, the association of aberrant CK2α expression with decreased patient disease-free and overall patient survival in many cancers [37] make this enzyme a promising theranostic target for cancer therapy [106,107]. Indeed, CK2 inhibition combined with several targeted agents has already been shown to be effective in different types of cancers [108,109,110,111,112]. ATM is central to the repair of DSBs in DNA, thereby controlling genome stability, cell survival and resistance to radio- and chemo-therapeutic treatment [113,114,115]. Over recent years, a wealth of evidence has accumulated showing that ATM is also part of many other signaling networks, including chromatin remodeling, cell metabolism and growth, senescence and notably, oxidative stresses in response to ROS generation [27,29,116,117,118,119]. ROS contain unpaired electrons allowing them to interact with DNA and other biomolecules, causing irreversible cellular damages. They may affect cancer cells in a dual fashion. On one hand, ROS production can lead to carcinogenesis, either by activation of several oncogenic pathways or through oncogenic mutations in DNA [120,121,122]. On the other hand, excessive ROS generation has been found to be positively correlated with cell cycle arrest and apoptosis [123,124] and to suppress distant metastasis in metastatic human melanoma cells [125]. Over the last decades, the clinical relevance of oxidative stress/ROS as a target in human malignancies has greatly increased and several successful chemotherapeutic approaches have been designed with the aim of increasing intracellular ROS levels to trigger irreversible cellular damages and apoptosis in tumor cells (reviewed in Reference [126]). As an example, sorafenib was shown to disrupt the mitochondrial membrane potential in RCC, leading to increased ROS and thus breaking resistance to TNF (Tumor necrosis factor) -related apoptosis inducing ligand (TRAIL)-induced apoptosis [127]. 

Here, we describe that as a single agent, CX-4945 moderately induced cell death in VHL-deficient renal cancer cells but unexpectedly, triggered a strong ATM upregulation, providing a potential link between CK2 and ATM pro-survival pathways [38]. In contrast, dual targeting of CK2 and ATM exhibited efficacy to promote vulnerability of RCC cells, impeding cell migration and inducing ROS-dependent apoptosis in VHL-deficient cells. Our drug combination demonstrated a greater anti-tumor activity than sunitinib and exhibited low toxicity in normal tissue/cells. The NOX4 isoform has been identified as a critical modulator of redox homeostasis in renal cells. We found that in response to ATM/CK2 blockade, both ROS production and cell apoptosis were significantly decreased in NOX4-knockout cells. Increasing evidence suggests that phosphorylation of various NOX proteins or their regulatory cofactors may play important roles in regulating the activity of these enzymes [128]. For instance, ATM inhibition was shown to increase NOX4 expression in normal fibroblasts [129]. Moreover, CK2 was described as a negative modulator of NOX4 [130]. Collectively, these data suggest that the ATM/CK2 kinases and NOX4-generated ROS might be functionally connected. ROS were also involved in the regulation of hypoxic and non-hypoxic induction of HIF-1 under various conditions [131,132]. Moreover, both ROS generation and NOX4 expression are essential for HIF-2α transcriptional activity in VHL-deficient renal cell carcinoma [133], indicating that higher levels of ROS can contribute to HIF-2α stabilization and are key to facilitate and sustain the aggressive phenotype of cancer cells [134]. We observed an enhanced HIF-2α expression in VHL^−^ 786-O MCTS upon treatment with each drug alone or in combination. Importantly, the sensitivity of 786-O VHL^−^ MCTS was strongly affected by genetic disruption of HIF-2α. Conversely, high HIF-2α expression in these cells appears as a key determinant of their response to combined CK2/ATM pathway inhibition, suggesting a functional link between CK2/ATM kinases and HIF-2α. This indicates that baseline level of HIF-2α expression might represent a potential efficacy biomarker predictive of response to our drug combination. HIF-2α drives the expression of multiple target genes with tumorigenic functions [5,8,12,135,136,137]. Recently, small molecules that directly inhibit HIF-2α were shown to cause tumor regression in preclinical models of primary and metastatic VHL-defective ccRCC [10,11,138,139]. However, VHL-defective ccRCC cell lines display unexpectedly variable sensitivity to HIF-2α-targeted therapies [11], which provide effective treatment for only a subset of patients [140]. Mechanistically, phosphorylation of HIF-1α has been demonstrated to enhance transactivation of target genes by either disrupting its interaction with VHL and thereby stabilizing HIF-1α, or by increasing the affinity of HIF-1α for transcriptional coactivators [141]. We showed that CK2 associates with and retains its catalytic activity towards HIF-2α, suggesting a functional link between them. Hypoxia-induced phosphorylation by CK2 has been reported in the c-TAD domain of HIF-2α (Thr844) and mutation of this residue decreased reporter activity, possibly by increasing HIF affinity for an enzyme known as Factor Inhibiting HIF (FIH) [69,71]. Finally, transcriptome profiling of VHL^−^ 786-O MCTS treated with our drug combination not only confirms our results but also opens perspectives to explore additional mechanisms driving apoptosis in VHL-deficient renal carcinoma cells upon cooperative blockade of CK2 and ATM kinases.

## 5. Conclusions

Genomic analyses have revealed relationships between synthetic lethal interactions and genetic lesions that may be exploited therapeutically [21]. Chemo-genetic screens revealed CK2 and ATM kinases as synthetic lethal targets in VHL-deficient renal carcinoma cells. This vulnerability was characterized using various models, including cells grown as monolayers, tumor spheroid cultures and tumor tissue slices from xenografts and clinical patient samples. Combined inhibition of CK2 and ATM eliminated VHL-deficient renal carcinoma cells by increasing NOX4-mediated ROS production. Importantly, HIF-2α acts as a key determinant that potentiates this response. Overall, given the demonstrated safety of CX-4945 in initial human studies, these preclinical data may justify the implementation of clinical trials using CX-4945 in combination with ATM inhibitors in a subset of HIF-2α-expressing VHL-defective ccRCC patients. Our findings have translational implications which are currently evaluated in the frame of the preclinical trial NCT03571438 (https://clinicaltrials.gov/ct2/show/NCT03571438). 

## 6. Patents

The data presented in this study forms a basis for a patent: 

PCT/EP2016/072458 (US Patent): A SYNTHETIC LETHAL DRUG COMBINATION FOR TREATING RENAL CELL CARCINOMA. Filhol O., Cochet C., Giacosa S., Pillet C., Barette C., Soleilhac E.

## Data Availability

The RNA-sequencing datasets accessed for this manuscript are available in the publicly accessible repository, at the ArrayExpress repository under the accession number E-MTAB-9977 (https://www.ebi.ac.uk/arrayexpress/experiments/E-MTAB-9977).

## Supplementary Materials

The following are available online at https://www.mdpi.com/2072-6694/13/3/576/s1, Figure S1: Flow chart of the screening, Figure S2: Cell viability of treated-786-O and RPTEC cells under normoxic and hypoxic conditions, Figure S3: Biochemical study of CK2–HIF-2α interaction, Figure S4: Images of ROS detection in treated 786-O MCTS, Figure S5: Western blot of 4 shNOX4 expressing 786-O VHL^−^ cells, Figures S6–S8: Analysis of treated spheroids transcriptomes, Table S1: Genes targeted by shRNA sequences, Table S2: Chemicals used in the screening, Table S3: Data produced by gene expression and gene ontology enrichment analyses of 786-O spheroids.
