# Supplementary material for: Cooperative Blockade of CK2 and ATM Kinases Drives Apoptosis in VHL-Deficient Renal Carcinoma Cells through ROS Overproduction
Source: Cancers (Basel). 2021 Feb 2;13(3):576. doi: 10.3390/cancers13030576 (PMC7867364; doi:10.3390/cancers13030576)
Supplement: Supplementary file 1 [file cancers-13-00576-s001.zip › cancers-1093644-supplementary/Supplementary material/supplemental legendes_Revised.docx]

**Supplemental Legends:**

**Figure S1**: Flow chart of the screening.

**Figure S2**: 786-O (**A,B**) or RPTEC (**C**) cells were culture under 20 % O_2_ (normoxia, **A**) or 1.5 % O_2_ (hypoxia, **B,C**) and treated with either vehicle, 2.5 µM or 5 µM of KU-60019 in combination with either vehicle or increasing concentrations of CX-4945, Vehicle (●), 1 µM (■), 2.5 µM (▲) or 5 µM (▼). Cell viability was measured and represented as a percentage compared to DMSO taken as 100 %.

**Figure S3: A:** A cell extract from HEK293T cells expressing HA-tagged HIF-2α was applied on a DEAE-chromatography column that was subjected to a NaCl gradient. CK2 activity was quantified in elution fractions that were further analyzed by western blot to detect both CK2 catalytic subunits and HIF-2α. **B:** Fractions 9-12 were incubated with [γ-^32^P]-ATP/MgCl_2_ in the absence (DMSO) or presence of CX-4945 and phosphorylated proteins were resolved on SDS-PAGE and detected by autoradiography. **C:** HIF-2α was immunoprecipitated from transiently transfected HEK293T cells. **a**) Beads were then resuspended in 20 μl of kinase buffer in the absence or presence of CX-4945. Kinase reactions were initiated by addition of 10 μCi [γ-^32^P]-ATP for 10 min at 20°C. Phosphorylated proteins were analyzed by SDS-PAGE and autoradiography. **b**) CK2 activity was assayed in HIF-2α immunoprecipitates, in the absence or presence of CX-4945, using the specific CK2 peptide substrate RRREDEESDDEE.

**Figure S4: A:** 786-O MCTS were treated for 24 h with either vehicle (DMSO, 0.15%), 5 µM of CX-4945 or 10 µM of KU-60019, or both, in the presence of MitoSOX (5 µM). ROS production was monitored using an Essen IncuCyte Zoom live-cell microscope. Images taken automatically every 1 h for 24 h, Bright field and fluorescent overlayed images show 786-O spheroids at times (0 h, 12 h, 18 h and 24 h). Scale bar 50 µm. **B:** Spheroids treated with KU-CX or DMSO were fixed and stained at 24 h and mounted in an agarose syringe for LSFM imaging. 3D views of spheroids treated for 24 h with DMSO (0.15 %) or KU-CX (10 µM and 5 µM respectively). Bleu = Hoechst ; Green = Phalloidin ; Red = MitoSox. Scale bar 450 µm.

**Figure S5: A:** Western blot of 4 shNOX4 expressing 786-O VHL- cells showing expression of NOX4 and GAPDH as loading control. **B**: Quantification of NOX4 expression relative to GAPDH (*n*=3).

**Figure S6: A:** Principal Component analysis (PCA) of transcriptomes quantified from spheroids of tumor cell line 786-0. The spheroids were treated with either a vehicle (DMSO: gray point), drugs alone (KU-60019: blue point; CX-4945: orange point) or in combination (KU + CX: purple point). **B:** Bar plot of the number of deregulated genes from the transcriptomes of spheroids treated with drugs alone (KU-60019: blue bar; CX-4945: orange bar) or in combination (KU + CX: purple bar). The genes were considered to be deregulated if the absolute value of their Log2 expression fold change, relative to their treatment by the vehicle (DMSO), is greater than 0.3. **C:** Bar plot of the number of significantly enriched Gene Ontology (GO) terms found by Gene Set Enrichment Analysis (GSEA) from deregulated genes in spheroids treated with CX-4945 drug alone (orange bar) or in combination (KU + CX: purple bar). No significantly enriched GO term was identified from genes deregulated in expression by the KU-60019 treatment. Two different GO resources were used to perform the statistical enrichment in terms: molecular functions (MF) and biological processes (BP). **D:** Enrichment map of GO BP terms found significantly enriched by GSEA from deregulated genes in spheroids treated with the drug combination KU + CX compared to DMSO. Terms are organized in a network with edges connecting overlapping gene sets. Thus, mutually overlapping gene sets tend to cluster together into functional module. The color gradient corresponds to the Normalized Enriched Score (NES) calculated by GSEA for each BP term enriched. A positive NES (red circle) indicates that the BP term is activated (ie enriched in genes overexpressed by the KU + CX combination), while a negative NES (blue circle) indicates that the BP term is repressed (ie enriched in genes underexpressed by KU + CX). The size of the circle indicates the number of genes annotated in the BP term. **E:** Bar plot of some core enriched BP terms shared by the spheroids treated by the CX-4945 drug alone or by the KU + CX combination. The NES of each shared BP term is colored according to the treatment: CX-4945 alone (orange bar) or KU + CX (purple bar). A positive NES indicates that the BP term is activated by the treatment, while a negative NES indicate its repression. **F:** GSEA enrichment plot of the BP term named apoptotic DNA fragmentation found specifically enriched in spheroids treated with the combination KU + CX. The top portion shows the running enrichment score (ES) for the gene set as the analysis walks down the ranked gene list. The score at the peak of the plot is the ES for the gene set. A positive ES indicates gene set enrichment at the top of the ranked gene list (i.e. genes over-expressed by KU + CX). The middle portion shows where the members of the gene set appear in the ranked list of genes. The bottom portion shows the value as the logarithm of the fold change, between expression levels in spheroids treated by KU + CX versus the vehicle (DMSO), used as gene ranking metrics.

**Figure S7: A:** Distribution of the Log2 gene expression fold change for spheroids treated with drugs alone (KU-60019: blue line; CX-4945: orange line) or in combination (KU + CX: purple line), relative to the treatment by the vehicle (DMSO). The density plot was generared using Kernel Density Estimation (KDE). **B:** Fold change plot showing deregulated gene number by the different treatments according to fold change, relative to the control treatment (DMSO). **C:** Enrichment map of GO MF (Molecular Function) terms found significantly enriched by GSEA from deregulated genes in spheroids treated with the drug combination KU + CX. Terms are organized in a network with edges connecting overlapping gene sets. Thus, mutually overlapping gene sets tend to cluster together into functional module. The color gradient corresponds to the Normalized Enriched Score (NES) calculated by GSEA for each MF term enriched. A positive NES (red circle) indicates that the MF term is activated (ie enriched in genes overexpressed by the KU + CX combination), while a negative NES (blue circle) indicates that the MF term is repressed (ie enriched in genes underexpressed by KU + CX). The size of the circle indicates the number of genes annotated in the MF term. **D:** Bar plot of some core enriched MF terms shared by the spheroids treated by the CX-4945 drug alone or by the KU + CX combination. The NES of each shared MF term is colored according to the treatment: CX-4945 alone (orange bar) or KU + CX (purple bar). A negative NES indicates that the MF term is repressed by the treatment.

**Figure S8: A:** Enrichment map of GO BP terms found significantly enriched by GSEA from deregulated genes in spheroids treated with the drug CX-4945 alone. The color gradient corresponds to the NES calculated by GSEA for each BP term enriched. A positive NES (red circle) indicates that the BP term is activated (ie enriched in genes overexpressed by CX), while a negative NES (blue circle) indicates that the BP term is repressed (ie enriched in genes underexpressed by CX). The size of the circle indicates the number of genes annotated in the BP term. **B:** Enrichment map of GO MF terms found significantly enriched by GSEA from deregulated genes in spheroids treated with the drug CX-4945 alone.

**Table S1 :** **Genes targeted by shRNA sequences** cloned in the pLKO1 vector Hpgk-puro-cMV-tGFP. Lentiviral particles were provided by Merck Sigma-Aldrich.

| Gene | Accession Number | Gene | Accession Number |
| --- | --- | --- | --- |
| PDK1 | NM_002610 | BCL2A1 | NM_004049 |
| PLK2 | NM_006622 | TP53 | NM_000546 |
| PNCK | NM_198452 | ATM | NM_000051 |
| HCK | NM_002110 | ATR | NM_001184 |
| NEK6 | NM_014397 | CHEK1 | NM_001274 |
| TRIB3 | NM_021158 | CHEK2 | NM_007194 |
| PCTK3 / CDK18 | NM_002596 | MAPK14 | NM_001315 |
| MET | NM_000245 | CDK6 | NM_001259 |
| MELK | NM_014791 | PTK2 | NM_005607 |
| AURKB | NM_004217 | SRC | NM_198291 |
| KIT | NM_000222 | PAX2 | NM_000278 |
| PRKCD | NM_006254 | CSF1R | NM_005211 |
| PXK | NM_017771 | FGFR1 | NM_015850 |
| PDGFRL | NM_006207 | CA9 | NM_001216 |
| LCK | NM_005356 | AURORA | NM_003600 |
| PIM2 | NM_006875 | FLT1 | NM_002019 |
| MAPK1 | NM_138957 | CSNK2A1 | NM_001895 |
| AURKB | NM_004217 | CSNK2B | NM_001320 |
| NOX4 | NM_001143837.2 |  |  |

| **Molecule** | **Target** | **Molecule** | **Target** | **Molecule** | **Target** | **Molecule** | **Target** |
| --- | --- | --- | --- | --- | --- | --- | --- |
| U0126 | MEK | PHA665752 | MET | CHIR 99021 | GSK3β | Lapatinib | EGFR |
| AG370 | PDGF R | AT9283 | Aurora A/B | Tyrphostin 25 | EGFR | TSU 68 | TGFβR/  FGFR1 |
| SP600125 | JUNK | TIE 2 | ANGR | ML-7 | MLCK | CP 690550 | JAK3 |
| Gefitinib | EGFR | GDC0941 | PI3K | Rapamycin | mTOR | STF 62247 | autophagy inducer |
| Sunitinib | VEGFR | Roxolitinib | JAK/STAT | OSI 930 | cKIT | Baraserbib | Aurora B |
| Apatinib | VEGFR | RG1462 | EGFR | KRN633 | VEGFR | AG 490 | JAK2 |
| Doxorubicin | Topoiso-merase | H89 | PKA | GSK 1838705 | IGFR | SU 4312 | FLK1 |
| Vemurafenib | ERK | Kempollone | GSK3β | TGFβ R inhib | TGFβR | Bosutinib | ABL |
| SB203580 | P38 MAPK | SU11274 | MET | KU-60019 | ATM | Pazopanib | VEGFR |
| LY294002 | PI3K | AG490 | EGFR | Piceatannol | SYK | AZ 960 | JAK |
| Indirubin | GSK3β | PF562271 | FAK | Roscovitine | CDK | CX-4945 | CK2 |
| PF2341066 | MET | Olaparib | PARP | Axitinib | VEGFR & PDGFR | Temsirolimus | mTOR |
| SXG523 | MET | Enzastaurin | PKC | Imatinib | ABL | Aurora 1 inhibitor | Aurora A |
| WP1130 | DUB/ABL | Tyrphostin 1 | CTRL inactif | INCB018424 | JAK/STAT | AG 126 | IRAK |
| Fasentin | GLUT1 | KN62 | CamK | Crenolanib | PDGFR α | GW 5074 | cRAF |
| YM155 | Survivin | BML257 | AKT | Saracatinib | SRC | Dasatinib | ABL |
| Lavendustin | EGFR | NVP-ADW742 | IGF1R | GSK1120212 | MEK | Sorafenib | VEGFR |
| Hypericin | PKC | MK1775 | WEE1 | PP1 | SRC | Cyt 387 | JAK |
| Y27632 | ROCK | KX2-391 | SRC | ZM 336372 | cRAF | 5FU | DNA |
| Paclitaxel | Tubulin | Perifosine | AKT | AZD 0530 | SRC/ABL | Tozasertib | Aurora A |

**Table S2 : Chemicals**

All compounds were dissolved in DMSO at a concentration of 10 mM. CX-4945 was synthesized at the Plateau Synthèse Organique, Département de Chimie Moléculaire, Université Grenoble Alpes, according to the method described by [1]. The chemichal library was composed of two commercial libraries, one from Selleckchem (Tyrosine kinase inhibitors) and one from Enzo (Screen-well kinase inhibitors that were complemented with other inhibitors Paclitaxel, 5FU, doxorubinin). FDA-approved drugs are in red.

1. Pierre F, Chua PC, O'Brien SE, Siddiqui-Jain A, Bourbon P, Haddach M, et al. Discovery and SAR of 5-(3-Chlorophenylamino)benzo[c][2,6]naphthyridine-8-carboxylic acid (CX-4945), the first clinical stage inhibitor of protein kinase CK2 for the treatment of cancer. Journal of medicinal chemistry. 2010; 54: 635-54.

**Table S3: Data produced by gene expression and gene ontology enrichment analyses of 786-O spheroids.**

**A: rlog_FC.** Gene expression matrix normalized by using the rlog transformation implemented in the DESeq2 package. Genes having across the samples a null expression variance or a sum of read counts less than 10 were previously filtered out. The Log2 gene expression fold change were calculated for spheroids treated by drugs alone (KU-60019 or CX-4945) or in combination (KU + CX), relative to spheroids treated by the vehicle (DMSO).

**B: GO-BP_KU-CX_vs_DMSO.** List of significantly enriched Gene Ontology – Biological Process terms found by GSEA (P.adjust < 0.25) from deregelated genes in spheroids treated with the KU + CX combination.

**C: GO-MF_KU-CX_vs_DMSO.** List of significantly enriched Gene Ontology – Molecular Function terms found by GSEA (P.adjust < 0.25) from deregelated genes in spheroids treated with the KU + CX combination.

**D: GO-BP_CX_vs_DMSO.** List of significantly enriched Gene Ontology – Biological Process terms found by GSEA (P.adjust < 0.25) from deregelated genes in spheroids treated by the CX-4945 drug alone.

**E: GO-MF_CX_vs_DMSO.** List of significantly enriched Gene Ontology – Molecular Function terms found by GSEA (P.adjust < 0.25) from deregelated genes in spheroids treated by the CX-4945 drug alone.
