# Supplementary material for: Cooperative Blockade of CK2 and ATM Kinases Drives Apoptosis in VHL-Deficient Renal Carcinoma Cells through ROS Overproduction
Source: Cancers (Basel). 2021 Feb 2;13(3):576. doi: 10.3390/cancers13030576 (PMC7867364; doi:10.3390/cancers13030576)
Supplement: Supplementary file 1 [file cancers-13-00576-s001.zip › cancers-1093644-supplementary/Supplementary material/Table S1.docx]

**Table S2: Targeted genes.**

| Gene | Accession Number | Gene | Accession Number |
| --- | --- | --- | --- |
| PDK1 | NM_002610 | BCL2A1 | NM_004049 |
| PLK2 | NM_006622 | TP53 | NM_000546 |
| PNCK | NM_198452 | ATM | NM_000051 |
| HCK | NM_002110 | ATR | NM_001184 |
| NEK6 | NM_014397 | CHEK1 | NM_001274 |
| TRIB3 | NM_021158 | CHEK2 | NM_007194 |
| PCTK3 / CDK18 | NM_002596 | MAPK14 | NM_001315 |
| MET | NM_000245 | CDK6 | NM_001259 |
| MELK | NM_014791 | PTK2 | NM_005607 |
| AURKB | NM_004217 | SRC | NM_198291 |
| KIT | NM_000222 | PAX2 | NM_000278 |
| PRKCD | NM_006254 | CSF1R | NM_005211 |
| PXK | NM_017771 | FGFR1 | NM_015850 |
| PDGFRL | NM_006207 | CA9 | NM_001216 |
| LCK | NM_005356 | AURORA | NM_003600 |
| PIM2 | NM_006875 | FLT1 | NM_002019 |
| MAPK1 | NM_138957 | CSNK2A1 | NM_001895 |
| AURKB | NM_004217 | CSNK2B | NM_001320 |
| NOX4 | NM_001143837.2 |  |  |

Table S1 : Genes targeted by shRNA sequences cloned in the pLKO1 vector Hpgk-puro-cMV-tGFP. Lentiviral particles were provided by Merck Sigma-Aldrich.
