# Supplementary material for: Cooperative Blockade of CK2 and ATM Kinases Drives Apoptosis in VHL-Deficient Renal Carcinoma Cells through ROS Overproduction
Source: Cancers (Basel). 2021 Feb 2;13(3):576. doi: 10.3390/cancers13030576 (PMC7867364; doi:10.3390/cancers13030576)
Supplement: Supplementary file 1 [file cancers-13-00576-s001.zip › cancers-1093644-supplementary/Supplementary material/Table S2-revised.docx]

| **Molecule** | **Target** | **Molecule** | **Target** | **Molecule** | **Target** | **Molecule** | **Target** |
| --- | --- | --- | --- | --- | --- | --- | --- |
| U0126 | MEK | PHA665752 | MET | CHIR 99021 | GSK3β | Lapatinib | EGFR |
| AG370 | PDGF R | AT9283 | Aurora A/B | Tyrphostin 25 | EGFR | TSU 68 | TGFβR/  FGFR1 |
| SP600125 | JUNK | TIE 2 | ANGR | ML-7 | MLCK | CP 690550 | JAK3 |
| Gefitinib | EGFR | GDC0941 | PI3K | Rapamycin | mTOR | STF 62247 | autophagy inducer |
| Sunitinib | VEGFR | Roxolitinib | JAK/STAT | OSI 930 | cKIT | Baraserbib | Aurora B |
| Apatinib | VEGFR | RG1462 | EGFR | KRN633 | VEGFR | AG 490 | JAK2 |
| Doxorubicin | Topoiso-merase | H89 | PKA | GSK 1838705 | IGFR | SU 4312 | FLK1 |
| Vemurafenib | ERK | Kempollone | GSK3β | TGFβ R inhib | TGFβR | Bosutinib | ABL |
| SB203580 | P38 MAPK | SU11274 | MET | KU-60019 | ATM | Pazopanib | VEGFR |
| LY294002 | PI3K | AG490 | EGFR | Piceatannol | SYK | AZ 960 | JAK |
| Indirubin | GSK3β | PF562271 | FAK | Roscovitine | CDK | CX-4945 | CK2 |
| PF2341066 | MET | Olaparib | PARP | Axitinib | VEGFR & PDGFR | Temsirolimus | mTOR |
| SXG523 | MET | Enzastaurin | PKC | Imatinib | ABL | Aurora 1 inhibitor | Aurora A |
| WP1130 | DUB/ABL | Tyrphostin 1 | CTRL inactif | INCB018424 | JAK/STAT | AG 126 | IRAK |
| Fasentin | GLUT1 | KN62 | CamK | Crenolanib | PDGFR α | GW 5074 | cRAF |
| YM155 | Survivin | BML257 | AKT | Saracatinib | SRC | Dasatinib | ABL |
| Lavendustin | EGFR | NVP-ADW742 | IGF1R | GSK1120212 | MEK | Sorafenib | VEGFR |
| Hypericin | PKC | MK1775 | WEE1 | PP1 | SRC | Cyt 387 | JAK |
| Y27632 | ROCK | KX2-391 | SRC | ZM 336372 | cRAF | 5FU | DNA |
| Paclitaxel | Tubulin | Perifosine | AKT | AZD 0530 | SRC/ABL | Tozasertib | Aurora A |

**Table S2: Chemicals**

All compounds were dissolved in DMSO at a concentration of 10 mM. CX-4945 was synthesized at the Plateau Synthèse Organique, Département de Chimie Moléculaire, Université Grenoble Alpes, according to the method described by [1]. The chemichal library was composed of two commercial libraries, one from Selleckchem (Tyrosine kinase inhibitors) and one from Enzo (Screen-well kinase inhibitors that were complemented with other inhibitors Paclitaxel, 5FU, doxorubicin). FDA-approved drugs are in red.

1. Pierre F, Chua PC, O'Brien SE, Siddiqui-Jain A, Bourbon P, Haddach M, et al. Discovery and SAR of 5-(3-Chlorophenylamino)benzo[c][2,6]naphthyridine-8-carboxylic acid (CX-4945), the first clinical stage inhibitor of protein kinase CK2 for the treatment of cancer. Journal of medicinal chemistry. 2010; 54: 635-54.
