# Supplementary figures and images for: Cooperative Blockade of CK2 and ATM Kinases Drives Apoptosis in VHL-Deficient Renal Carcinoma Cells through ROS Overproduction
Source: Cancers (Basel). 2021 Feb 2;13(3):576. doi: 10.3390/cancers13030576 (PMC7867364; doi:10.3390/cancers13030576)

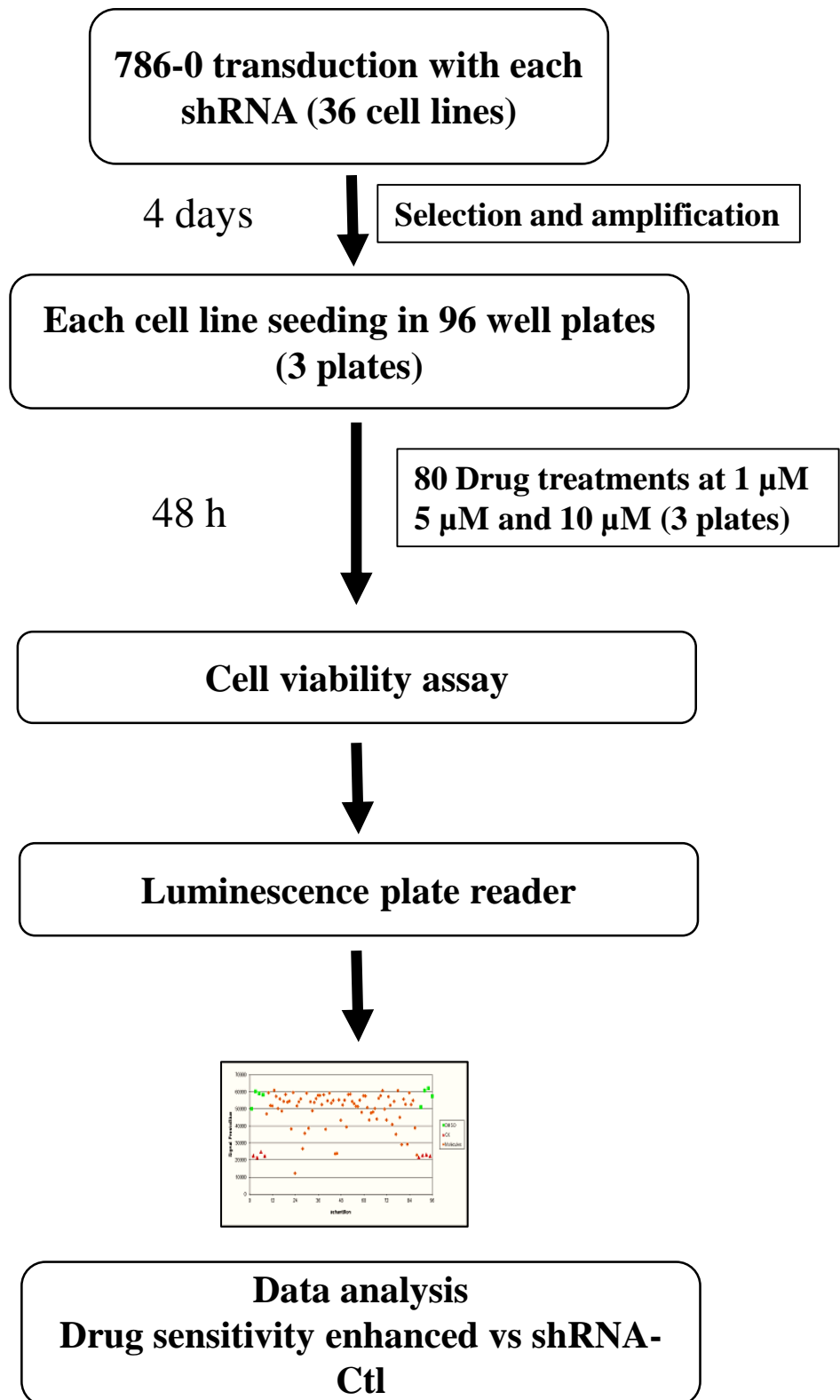

**Figure S1:** Flow chart of the screening.

Supplement: Supplementary file 1 [file cancers-13-00576-s001.zip › cancers-1093644-supplementary/Supplementary material/Supp Fig.1.pdf]

Figure S2

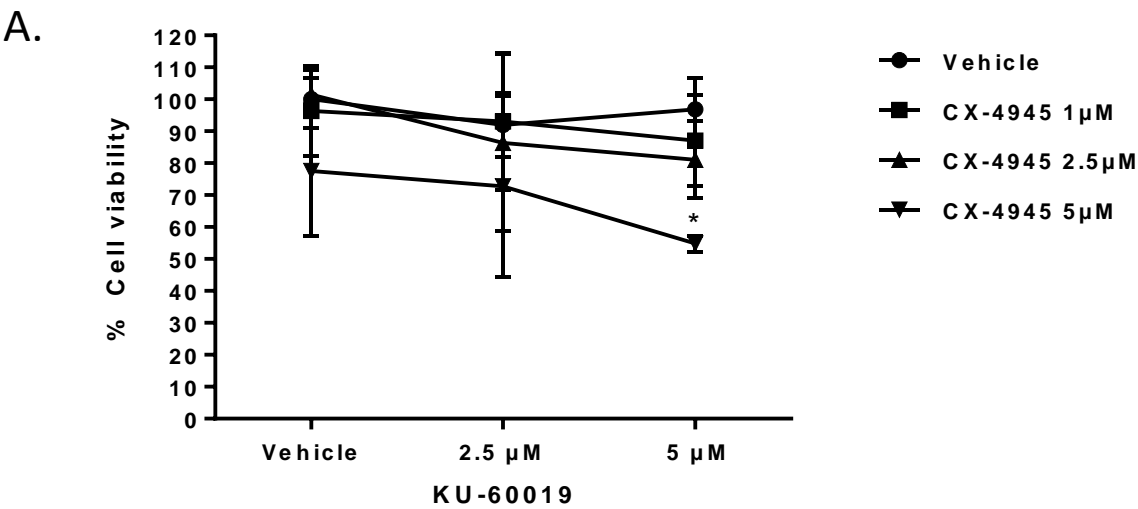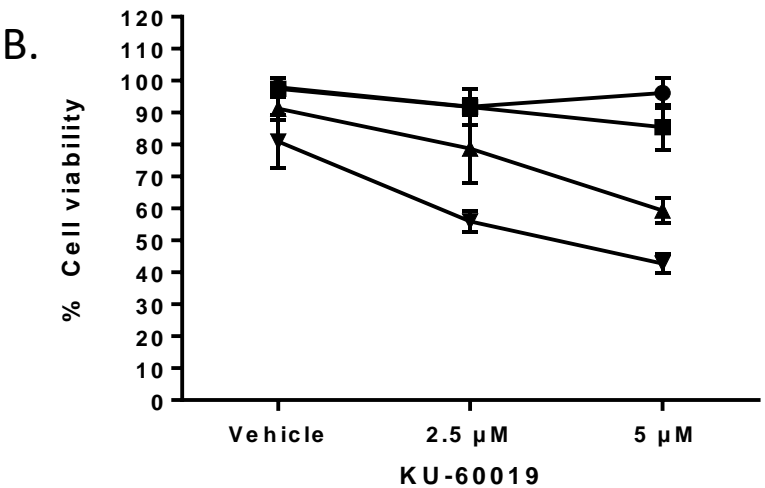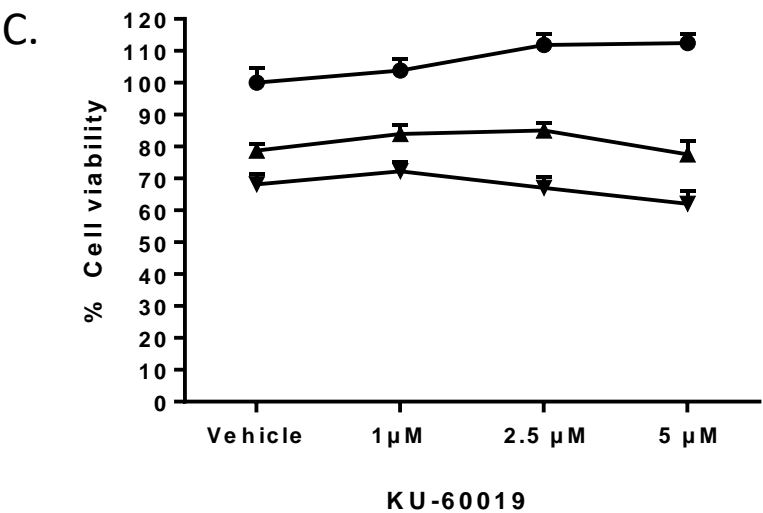

Supplement: Supplementary file 1 [file cancers-13-00576-s001.zip › cancers-1093644-supplementary/Supplementary material/Supp Fig.2.pdf]

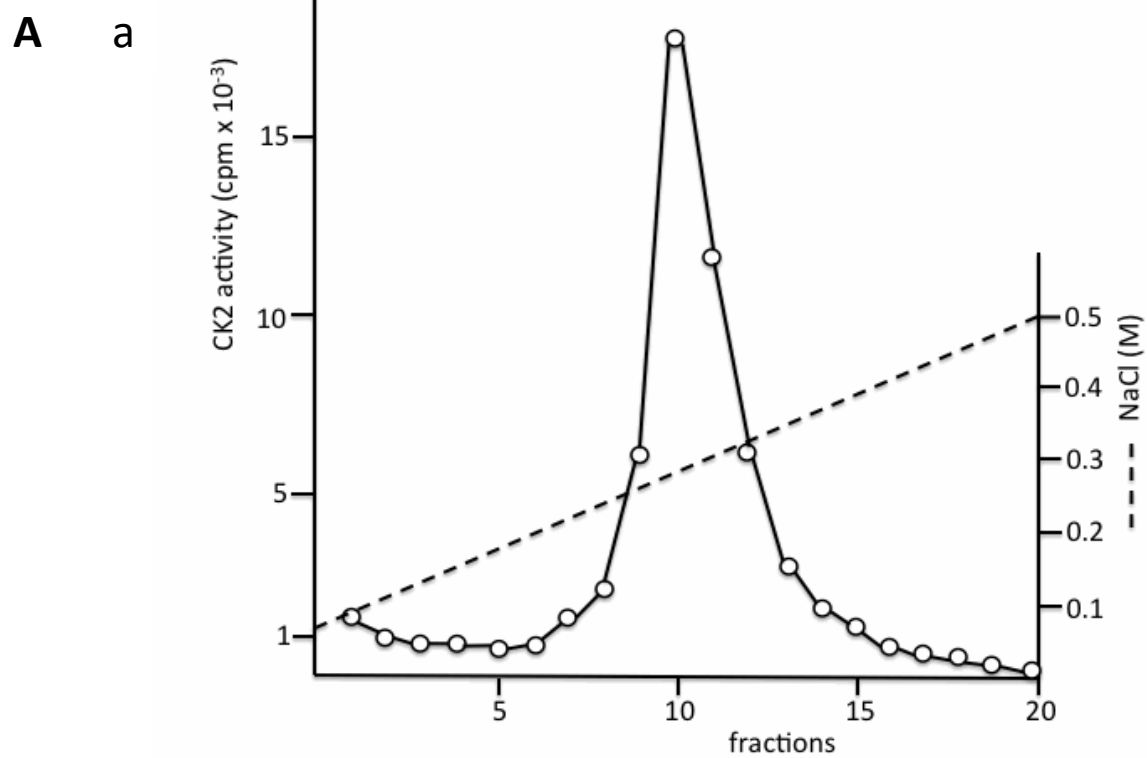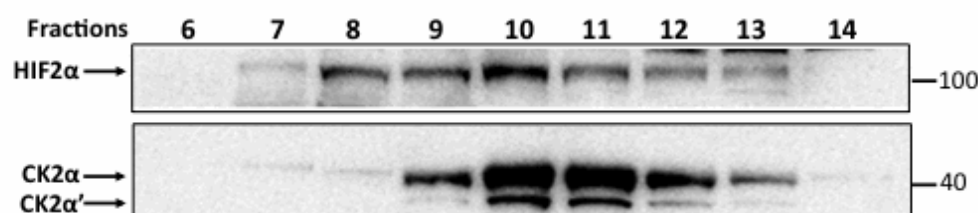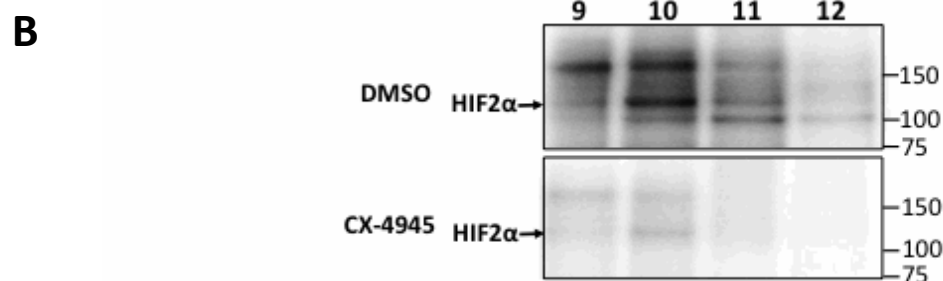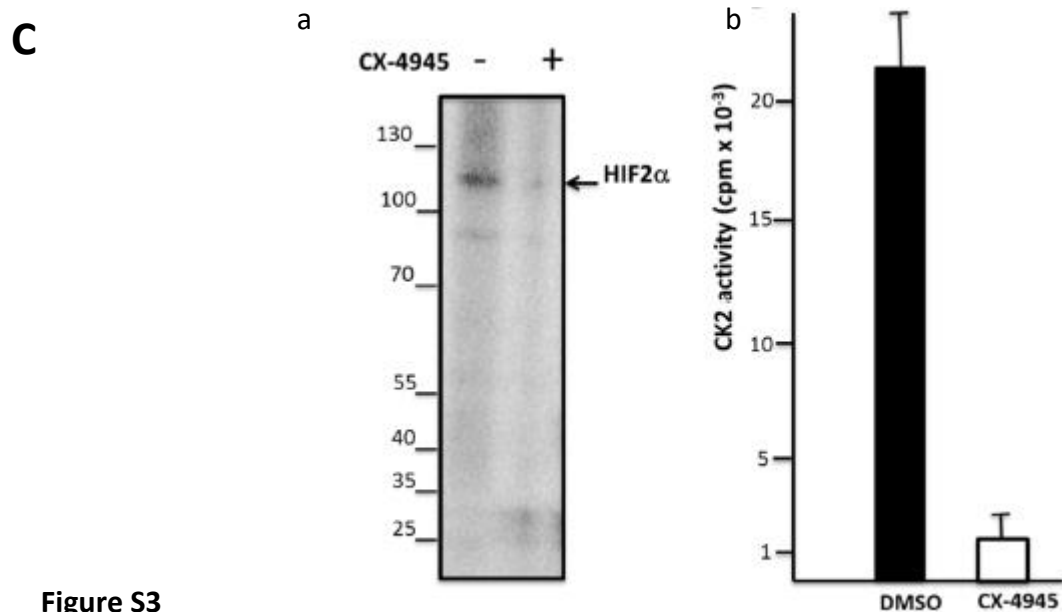

Figure S3

Supplement: Supplementary file 1 [file cancers-13-00576-s001.zip › cancers-1093644-supplementary/Supplementary material/Supp Fig.3.pdf]

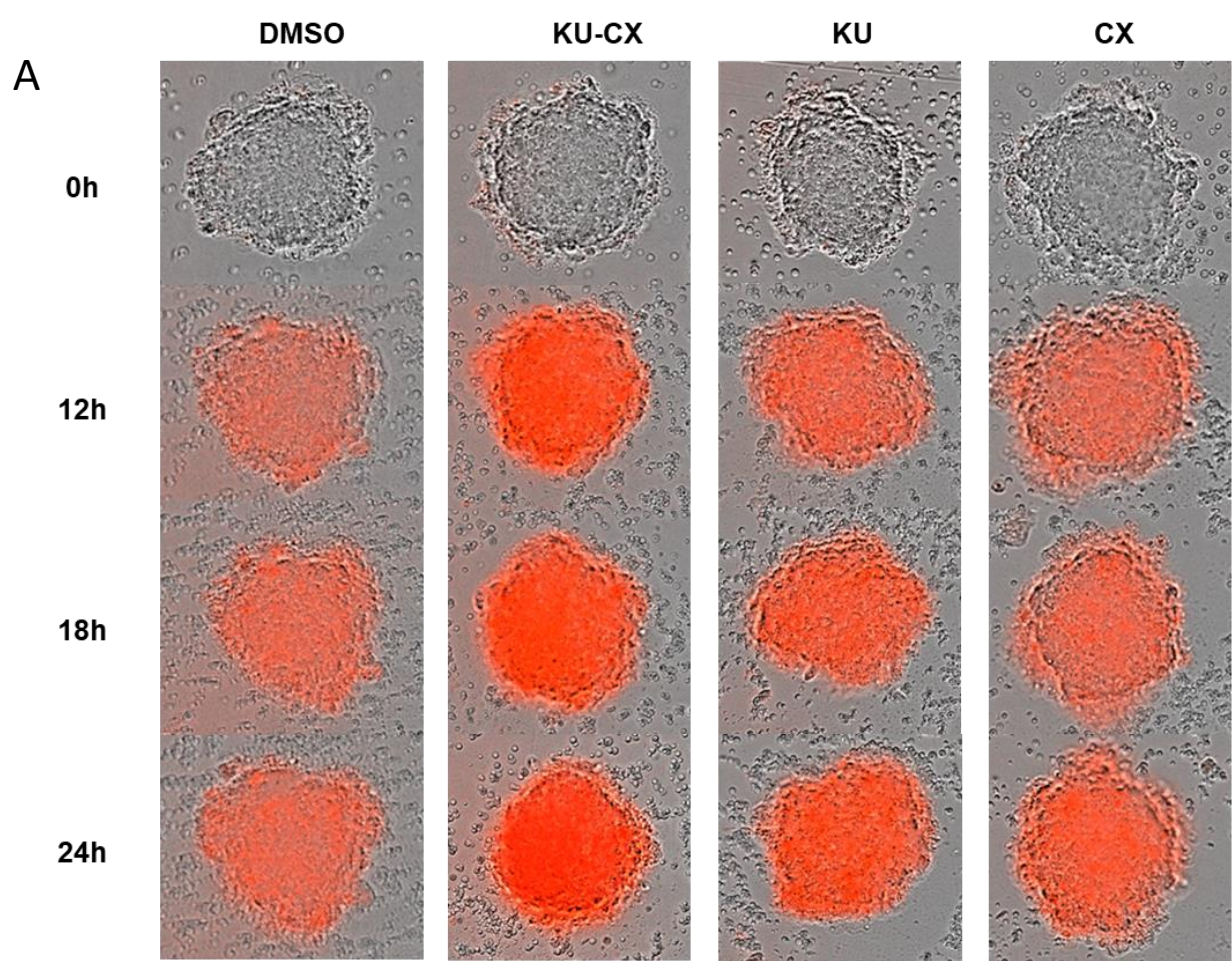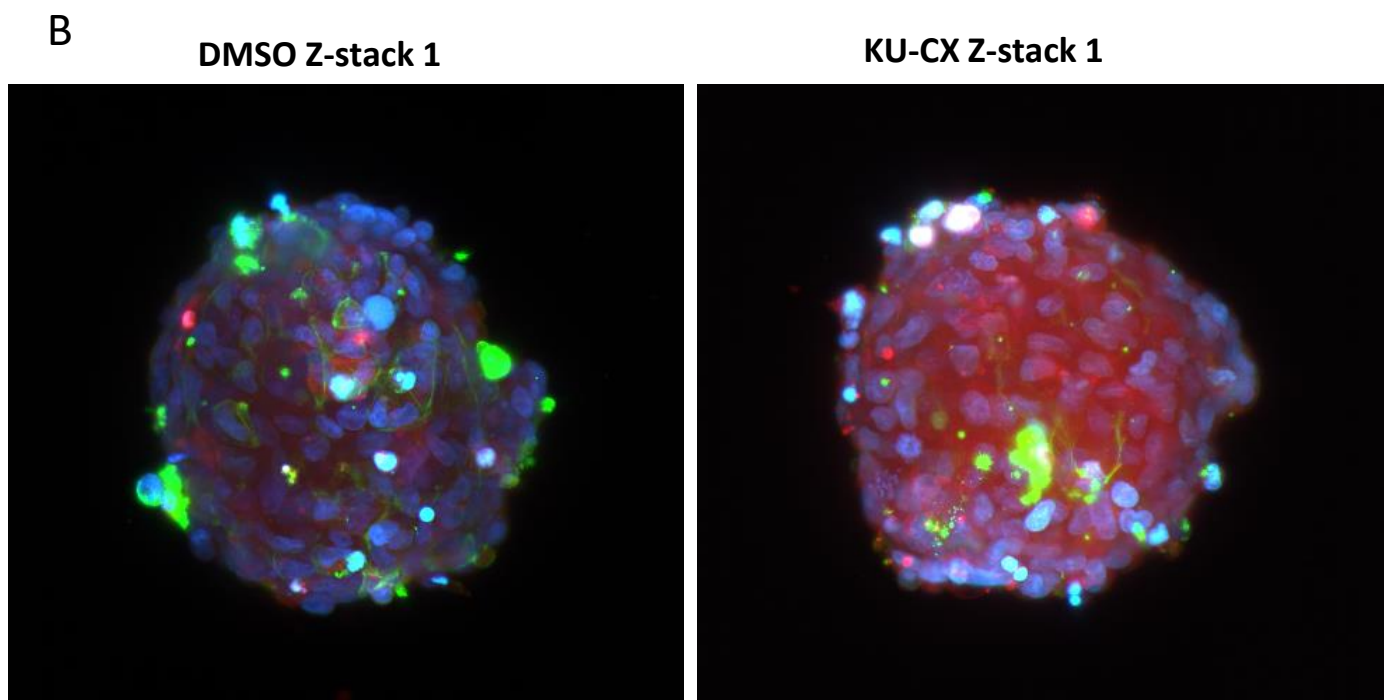

Figure S4

Supplement: Supplementary file 1 [file cancers-13-00576-s001.zip › cancers-1093644-supplementary/Supplementary material/Supp Fig.4.pdf]

A

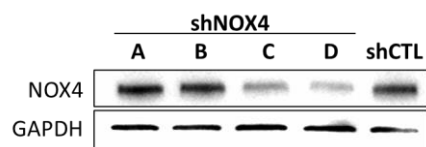

B

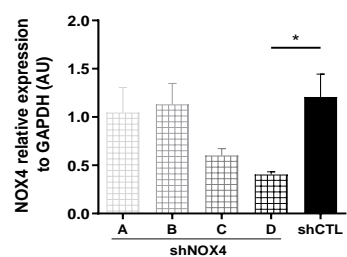

Figure S5

Supplement: Supplementary file 1 [file cancers-13-00576-s001.zip › cancers-1093644-supplementary/Supplementary material/Supp Fig.5.pdf]

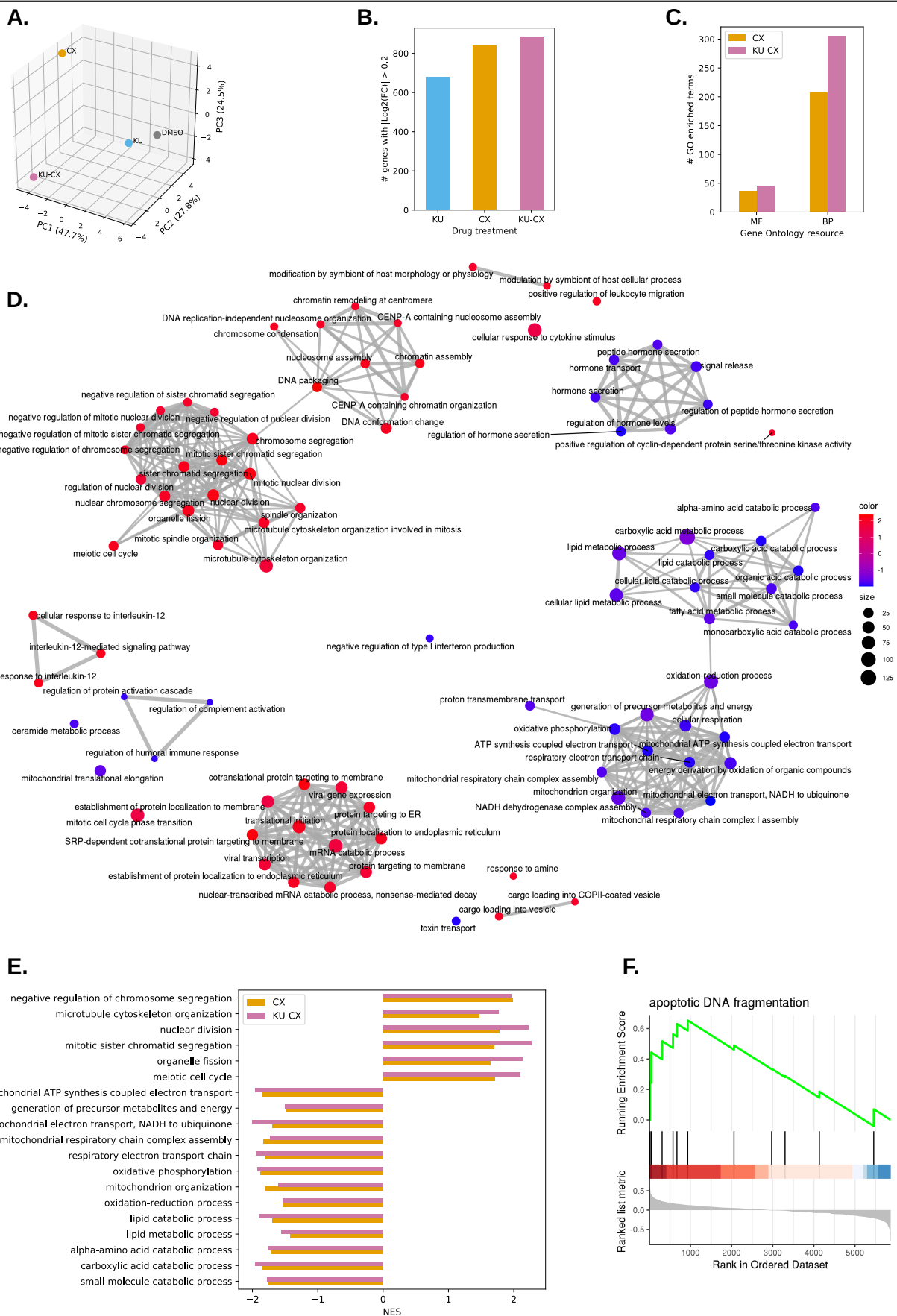

Figure S6

Supplement: Supplementary file 1 [file cancers-13-00576-s001.zip › cancers-1093644-supplementary/Supplementary material/Supp Fig.6.pdf]

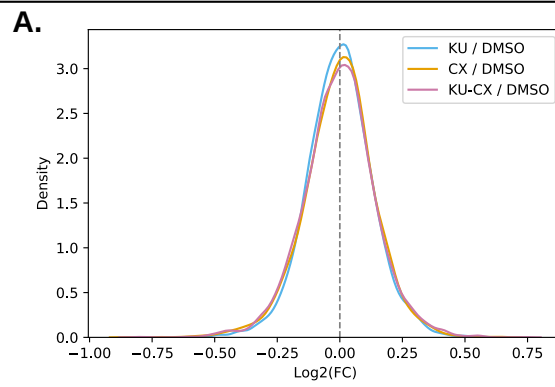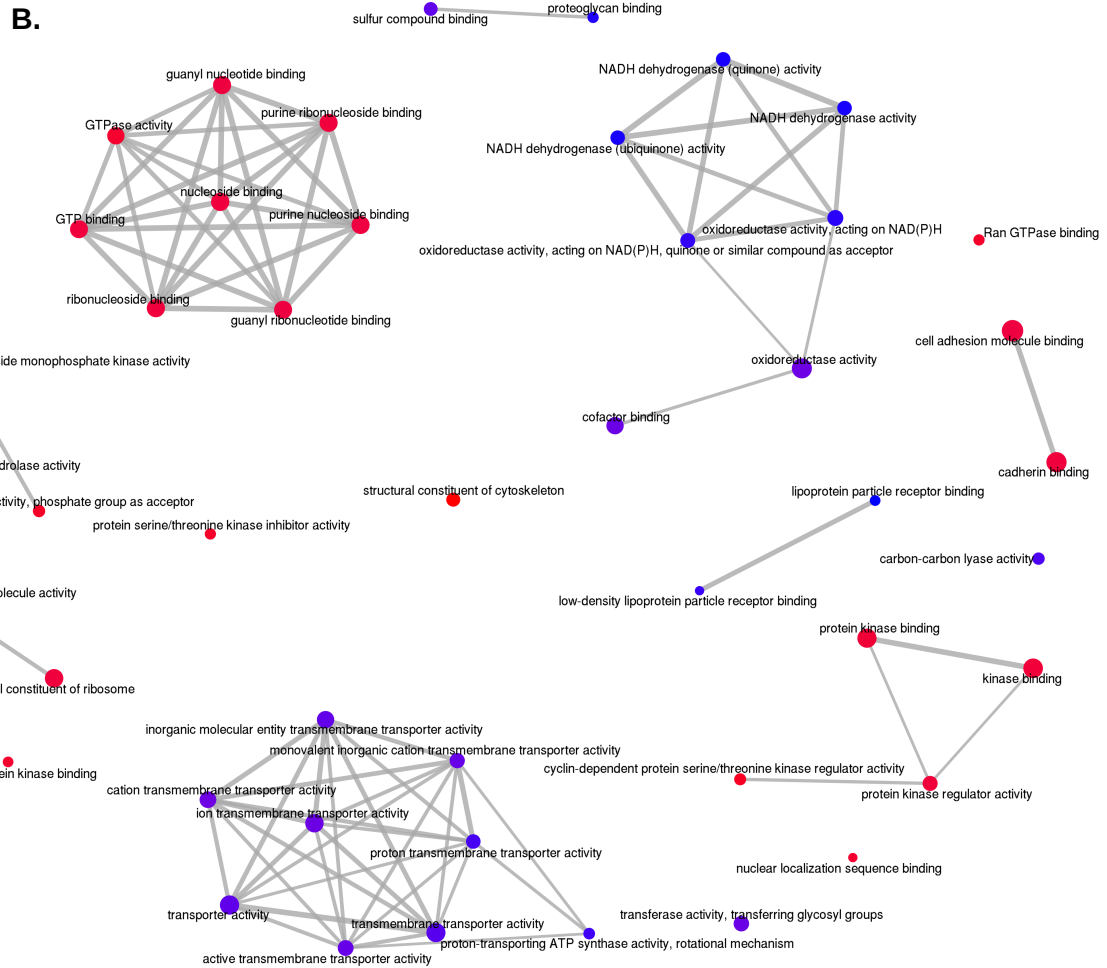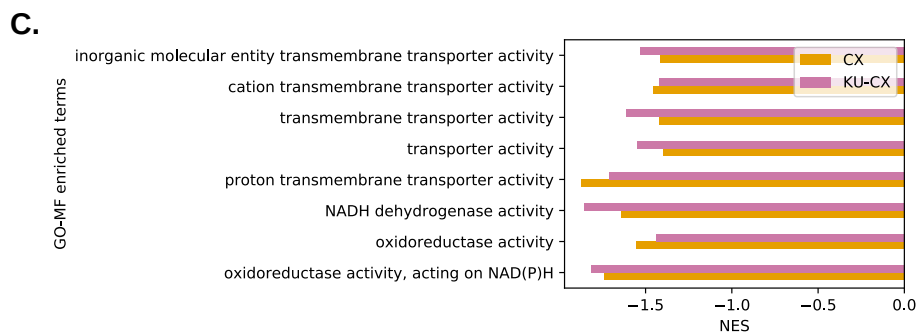

Figure S7

Supplement: Supplementary file 1 [file cancers-13-00576-s001.zip › cancers-1093644-supplementary/Supplementary material/Supp Fig.7.pdf]

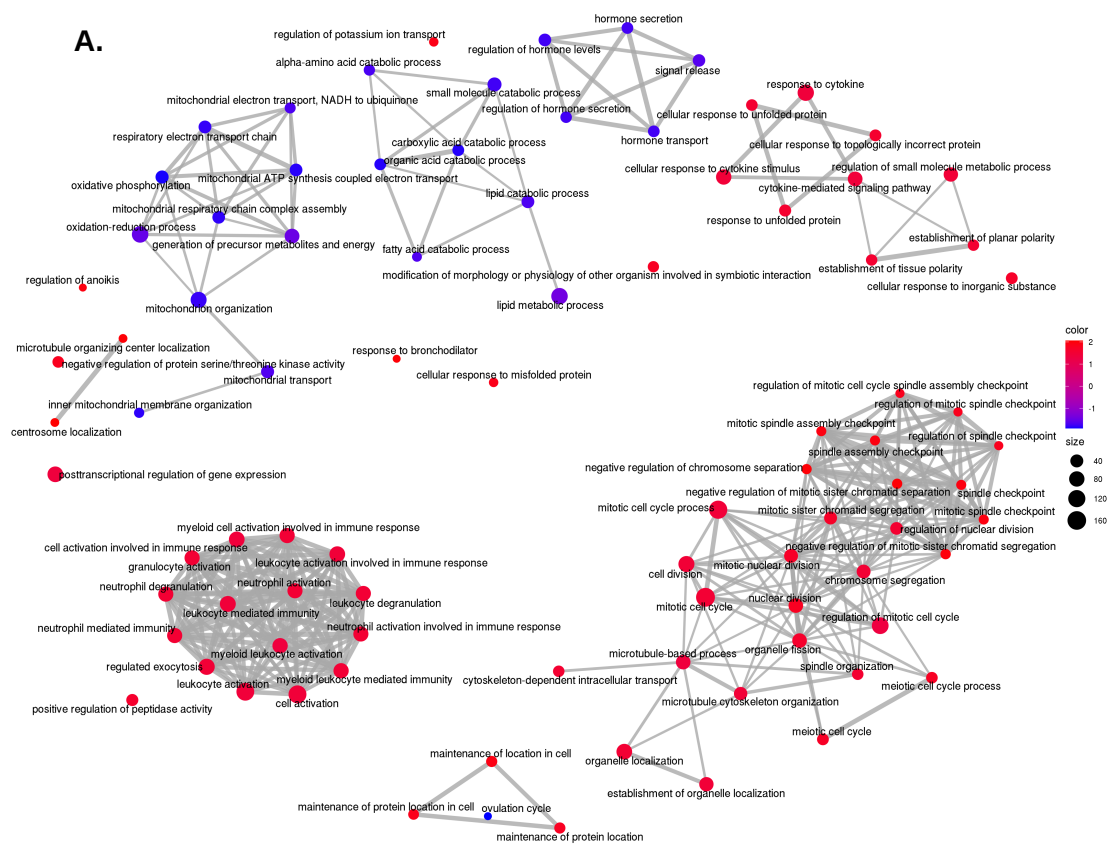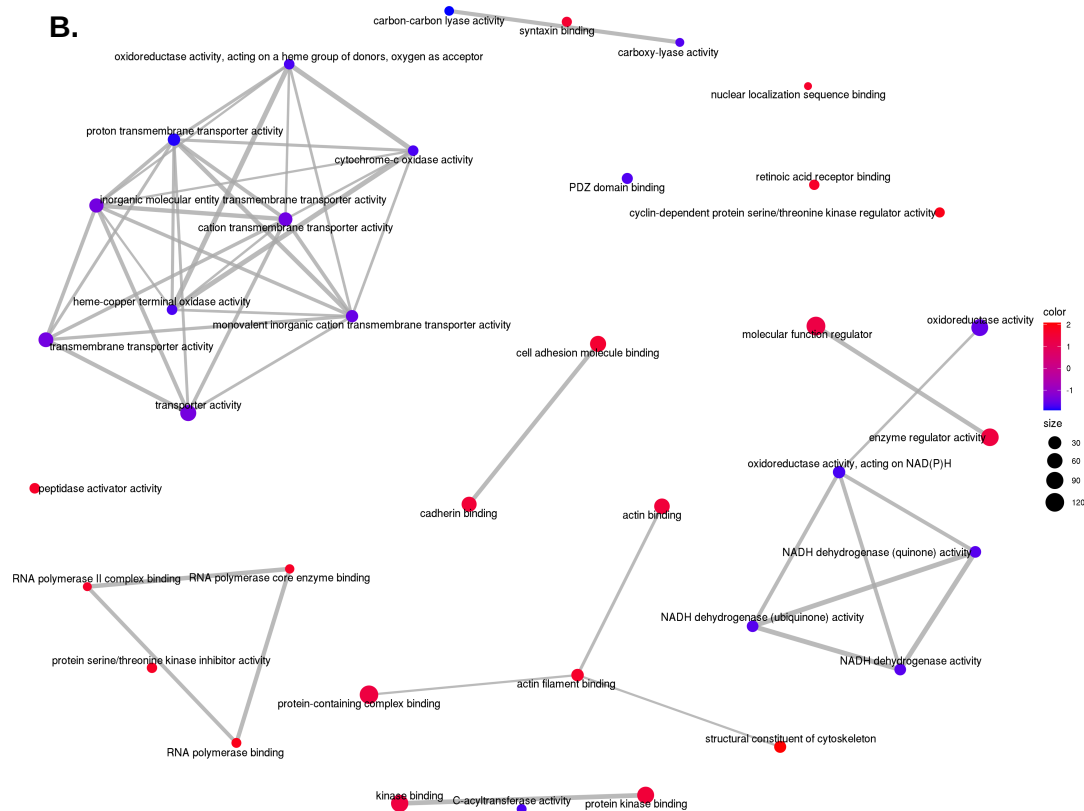

Figure S8

Supplement: Supplementary file 1 [file cancers-13-00576-s001.zip › cancers-1093644-supplementary/Supplementary material/Supp Fig.8.pdf]

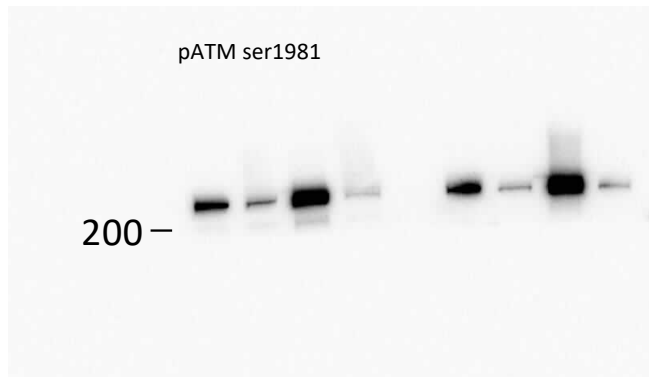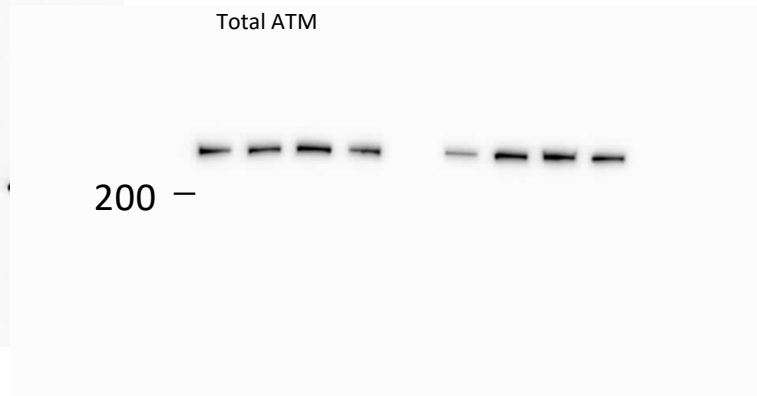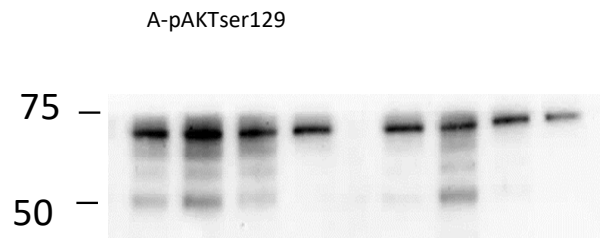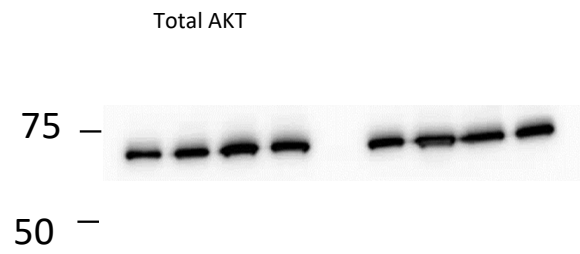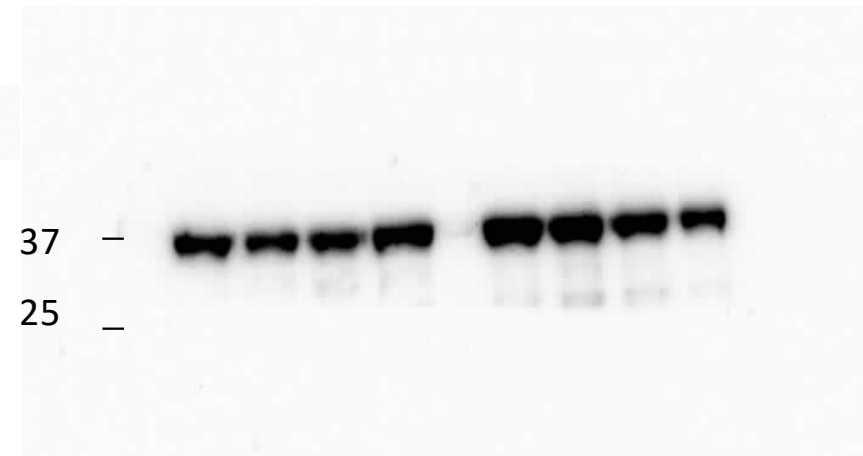

GAPDH

Figure 1

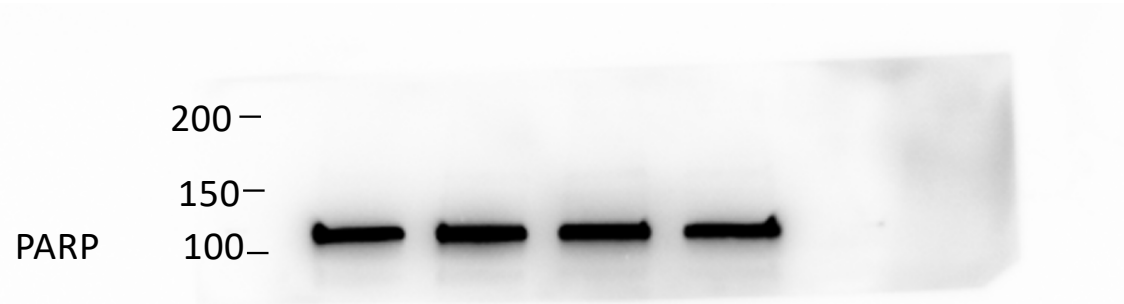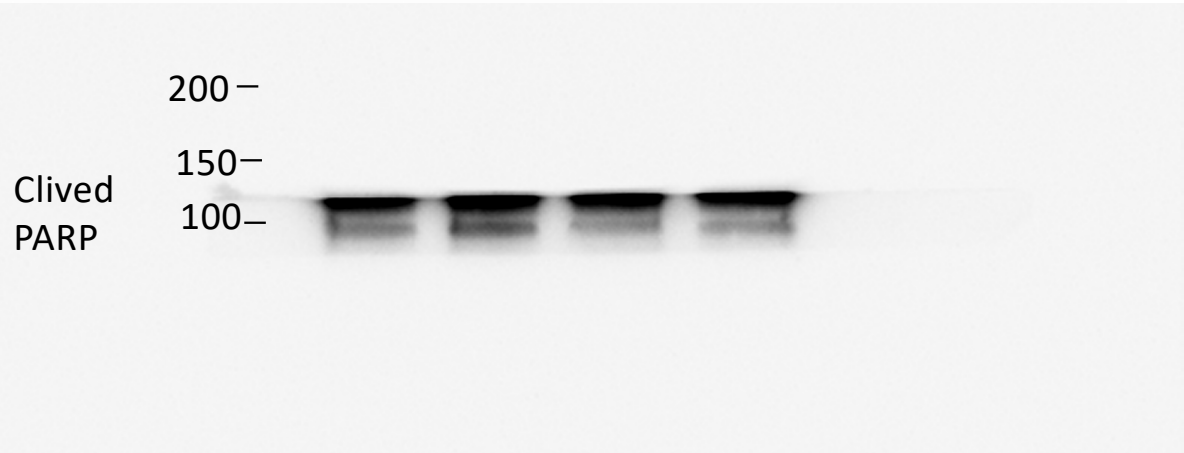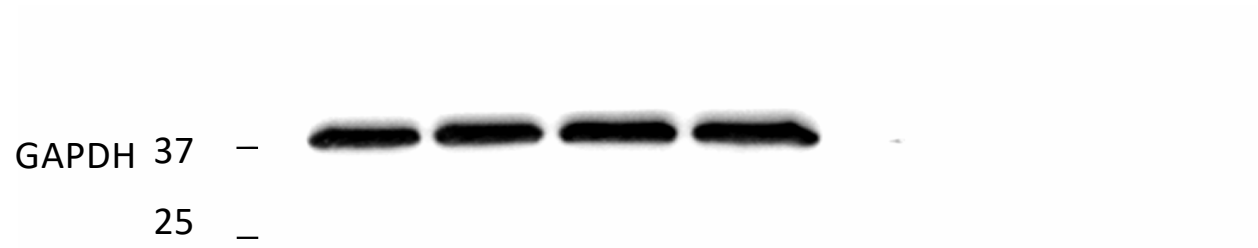

Figure 3

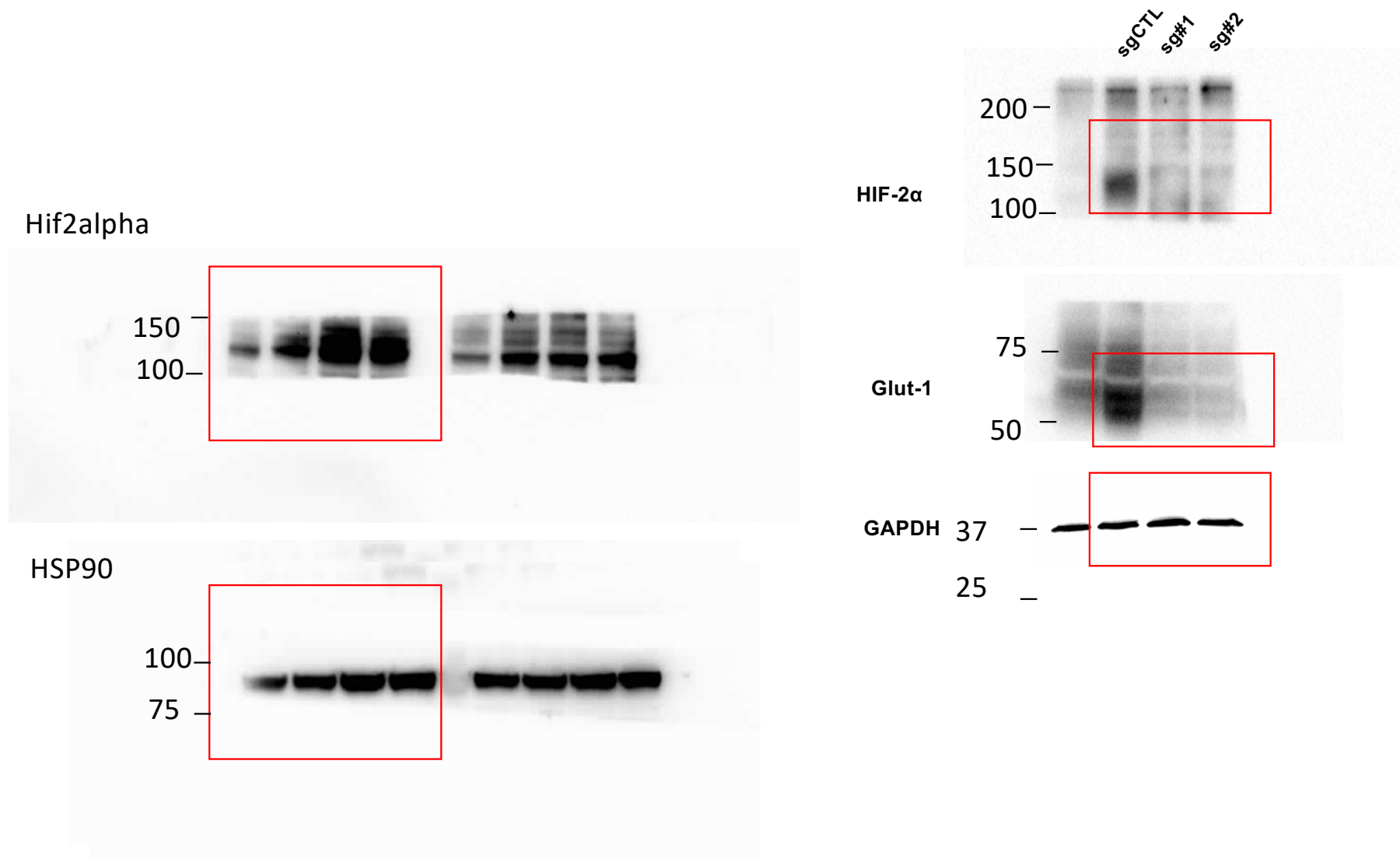

Figure 4

MDC1

200—  
150—  
100—

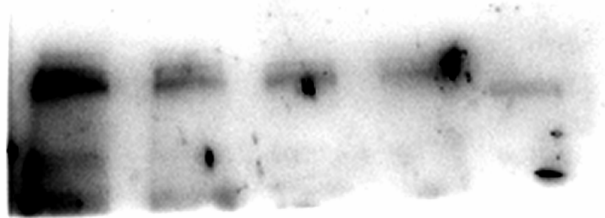

NOX4

75 —  
50 —

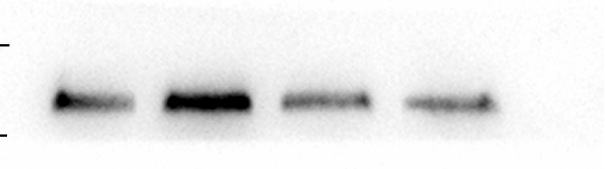

GAPDH

37 —  
25 —

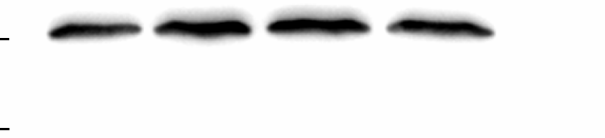

SOD1

25 —  
20 —  
15 —  
10 —

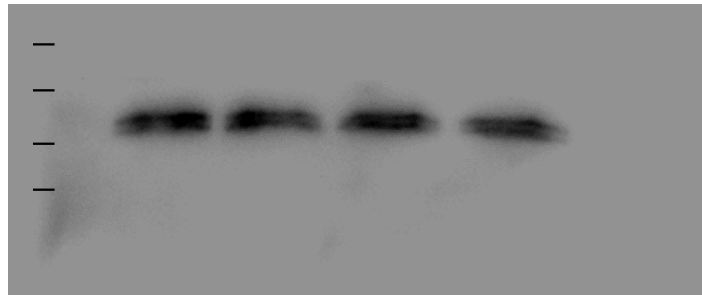

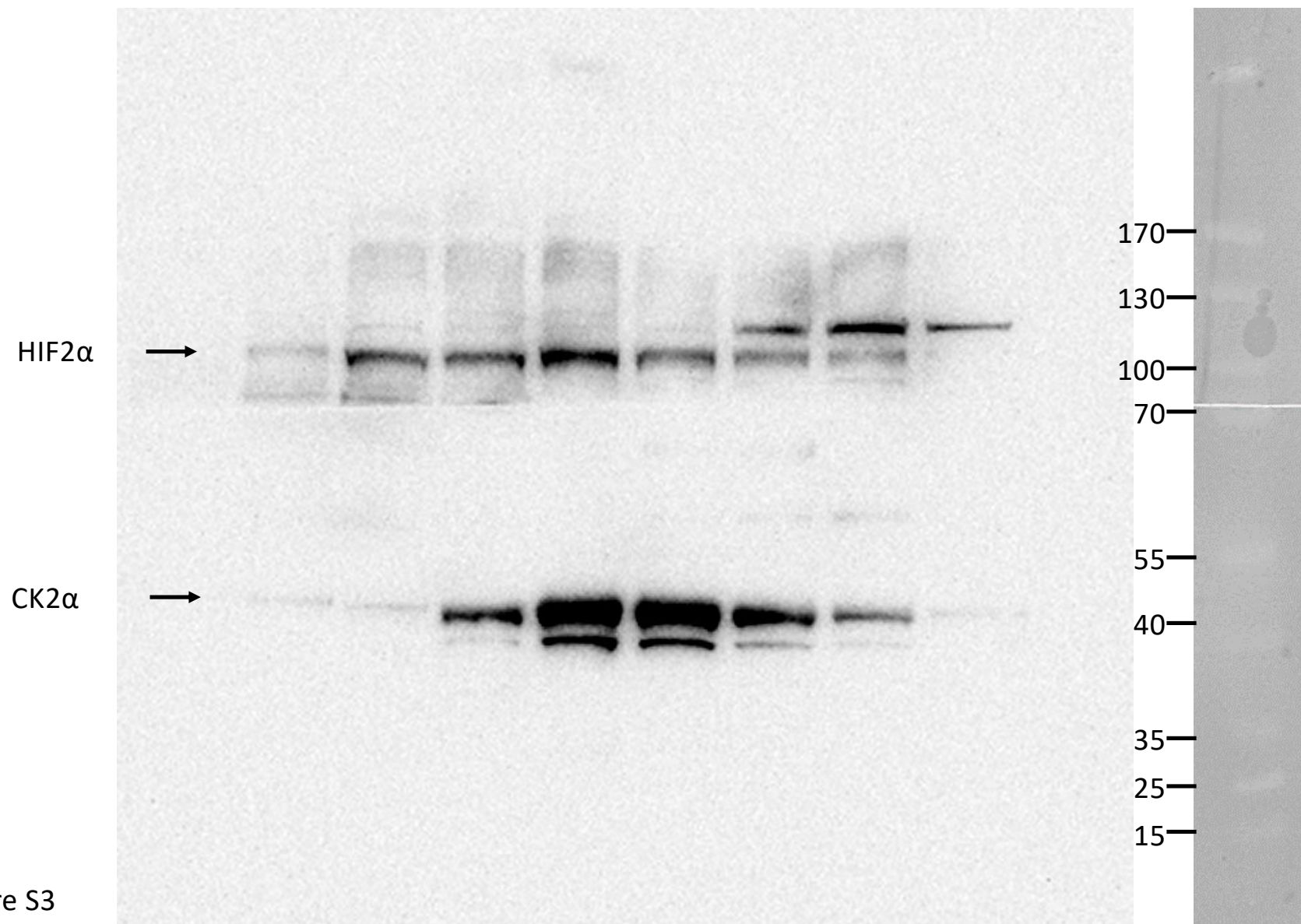

Figure S3

|               |   |   |   |   |   |   |   |   |   |   |
|---------------|---|---|---|---|---|---|---|---|---|---|
| HIF2 $\alpha$ | + | - | + | + | + | + | - | + | + | + |
| ATM           | - | + | + | - | - | - | - | - | - | - |
| CK2           | - | - | - | - | - | - | + | + | - | - |
| CX-4945       | - | - | - | - | + | - | - | - | + | - |

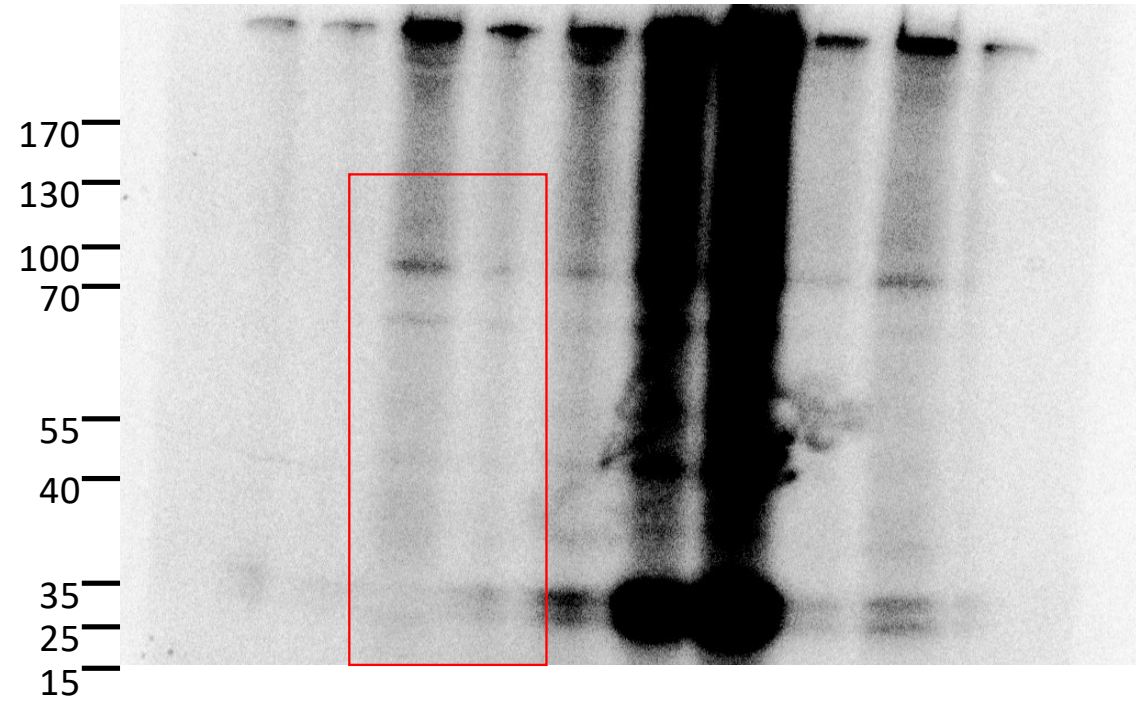

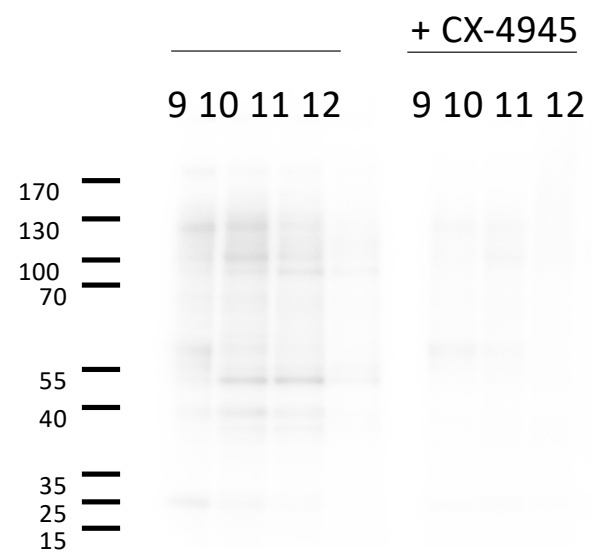

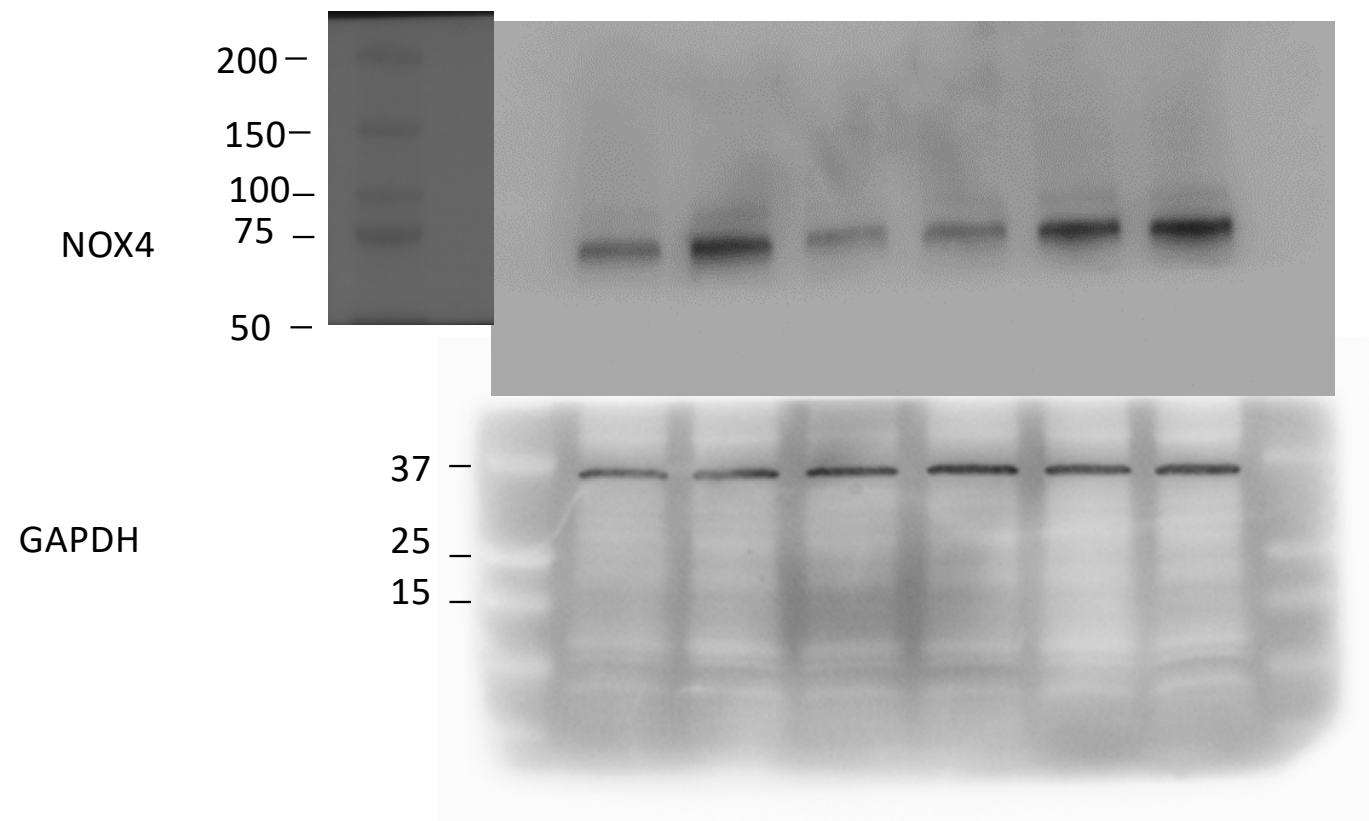

Supplement: Supplementary file 1 [file cancers-13-00576-s001.zip › cancers-1093644-supplementary/Supplementary material/whole western blot figures.pdf]
